# The Implementation Success of Technology-Based Counseling in Dementia Care: Scoping Review

**DOI:** 10.2196/51544

**Published:** 2024-01-25

**Authors:** Dorothee Bauernschmidt, Janina Wittmann, Julian Hirt, Gabriele Meyer, Anja Bieber

**Affiliations:** 1 Institute of Health and Nursing Science University Medicine Halle Martin Luther University Halle-Wittenberg Halle (Saale) Germany; 2 Center for Dementia Care, Institute of Nursing Science Department of Health Eastern Switzerland University of Applied Sciences St Gallen Switzerland; 3 Department of Clinical Research University Hospital Basel University of Basel Basel Switzerland

**Keywords:** implementation success, implementation outcomes, counseling, technology, dementia

## Abstract

**Background:**

Implementing technology-based counseling as a complex intervention in dementia care poses challenges such as adaptation to stakeholders’ needs and limited resources. While studies have examined the effectiveness of technology-based counseling, its successful implementation remains largely unexplored.

**Objective:**

We aimed to review the knowledge about the implementation success of technology-based counseling interventions for people with dementia and their informal caregivers.

**Methods:**

We conducted a scoping review and systematically searched CINAHL, the Cochrane Library including the Cochrane Central Register of Controlled Trials, MEDLINE, PsycINFO, and Web of Science Core Collection databases (April 2021) in combination with citation searching and web searching (November 2021). Studies reporting on technology-based counseling interventions for people with dementia or their informal caregivers were included, irrespective of the design. We used the conceptual framework for implementation outcomes to operationalize *implementation success* and applied the outcomes acceptability, adoption, appropriateness, feasibility, fidelity, implementation cost, penetration, and sustainability as categories to inform data extraction. We identified dimensions within the categories and synthesized results narratively and graphically.

**Results:**

We included 52 publications reporting on 27 technology-based counseling interventions. The studies were conducted in 9 countries and published between 1993 and 2021. As the design of the included studies varied, the number of participants and the type of data reported varied as well. The intervention programs were heterogeneous and ranged from single counseling interventions (such as helpline services) to counseling as part of a multicomponent program. Telephone, email, videoconferencing, social media (respectively chats), and web-based platforms were used for delivering counseling. We found data on appropriateness for all interventions and data on acceptability for most interventions, describing aspects such as consumer-perceived usefulness and helpfulness of services, as well as satisfaction. Information on the other categories of adoption, feasibility, fidelity, implementation cost, penetration, and sustainability was fragmented.

**Conclusions:**

The scope and depth of information on conceptual categories of the implementation success of technology-based counseling for people with dementia and informal caregivers varied. The data only partially covered the concept of *implementation success*, which highlights the need for a systematic evaluation accompanying the implementation. The application of theoretical approaches for implementation and adherence to the framework for developing and evaluating complex interventions are required to promote the implementation of complex interventions and to comprehensively assess implementation success.

**Trial Registration:**

PROSPERO CRD42021245473; https://www.crd.york.ac.uk/prospero/display_record.php?RecordID=245473

## Introduction

During the course of dementia, people with dementia, their families, and informal caregivers may need professional support to deal with the consequences of the disease [1-4]. Supportive interventions [5] are complex in their development, delivery, and impact, as they involve multiple components, aim at multiple outcomes, and are delivered in different settings. In addition, a broad range of skills are required of those who deliver the interventions [6] in dementia care.

Counseling for people with dementia and their informal caregivers is a supportive intervention that can be defined as conversational therapy in which a trained therapist listens to the person, enhances the individual’s ability to cope with the effects of dementia, and provides information and education [7,8]. Counselors provide information about the disease and support service options, offer the opportunity to share and discuss feelings or problems, and can enhance problem-solving and coping skills [9,10]. In the context of dementia, counseling services provided by professionals and tailored to individuals aim at various outcomes, such as reducing depressive symptoms and perceived burden, improving quality of life and self-efficacy, and encouraging the uptake of support services in the community [11]. Counseling interventions are therefore complex interventions comprising several components, such as specific training for providers, tools and instruments to individualize services, or different ways to access the available services [9,11]. Information and communication technologies may improve accessibility to counseling [12,13], and this aspect gained importance during the COVID-19 pandemic when remote counseling offered the possibility of providing support without the risk of infection [10,14].

To successfully translate complex interventions into practice, various challenges need to be overcome. Implementation can be defined as an “actively planned and deliberately initiated effort with the intention to bring a given intervention into policy and practice within a particular setting” [15]. The challenges described in implementing eHealth interventions in dementia care include adapting the interventions to match the skills and abilities of the target population, achieving user friendliness in the context of rapidly evolving technology, and addressing users’ concerns about security issues, especially when personal information is disclosed [16]. In implementing organizations, the lack of staff resources or the lack of interaction with staff as well as the reluctance of the provider to use the technology may impede implementation [16]. Furthermore, financial and time constraints can also act as barriers to implementation [16]. In the wider context, aspects such as stakeholders’ limited capabilities to support innovation or preferences for classically delivered care were identified as significant barriers [16].

Another challenge in the implementation of complex interventions is to operationalize and measure the success or effectiveness of implementation efforts. In a Cochrane Review on remotely delivered information, training, and support (including counseling) for informal caregivers of people with dementia, the authors found information on various aspects indicating implementation success such as acceptability, user satisfaction, or fidelity of implementation. As the indicators and assessment areas varied substantially across studies, data synthesis could not be conducted [9]. There are theoretical approaches that facilitate a comprehensive evaluation of implementation efforts [15]. One of them is the conceptual framework for implementation outcomes introduced by Proctor et al [17], which provides implementation-specific outcomes for assessing implementation success.

While there are studies investigating the effectiveness of technology-based counseling [8,9,11], the extent to which these interventions are successfully implemented remains unexplored. To address this gap in knowledge, we aimed to review the evidence and pursue the question of what is known about the implementation success of technology-based counseling interventions for people with dementia or their informal caregivers.

## Methods

### Design

According to the methodological approach of scoping reviews [18], we aimed at mapping evidence of implementation success to provide a comprehensive overview. We followed the methodological guidance [18] of the Joanna Briggs Institute and structured our report according to the PRISMA-ScR (Preferred Reporting Items for Systematic Reviews and Meta-Analyses extension for Scoping Reviews) [19].

### Protocol and Registration

This scoping review is embedded in the project “Technology-based counselling in dementia (TeCoDem),” for which a protocol has been developed [7] and registered with the international prospective register of systematic reviews (PROSPERO CRD42021245473; see the section *Deviations From the Protocol*).

### Eligibility Criteria

We included studies, irrespective of their design in English and German, that reported on technology-based counseling interventions for people with any type and severity of dementia or their informal caregivers. Interventions had to be tailored to individuals and provided remotely by professionals using various information technologies. Studies on people with mild cognitive impairment as well as studies on standardized interventions, such as cognitive behavioral therapy, on genetic counseling, and on counseling regarding diagnostics or screening for dementia were excluded. We also excluded studies describing interventions that focus mainly on care coordination or case management. Furthermore, publications reporting exclusively on the development of interventions were excluded [7].

### Information Sources and Search Strategy

We searched CINAHL, the Cochrane Library including the Cochrane Central Register of Controlled Trials (CENTRAL), MEDLINE via PubMed, PsycINFO via Ovid, and the Web of Science Core Collection databases (last search: April 22, 2021) without any filters and limiters. We systematically developed a search strategy that contained 3 components: *dementia*, *technology*, and *counseling*. Corresponding search terms and synonyms (eg, dementia/Alzheimer, technology/electronic, counselling/counseling/consultation) were identified through an orienting search using MEDLINE via PubMed, and we checked entry terms given in the Medical Subject Headings browser. The strategy was peer-reviewed by applying the Peer Review of Electronic Search Strategies [20]. In addition, we performed forward and backward citation searches of included studies and pertinent reviews via Scopus (last search: October 7, 2021) and a web search via Google and Google Scholar (last search: November 26, 2021) [21,22]. Full database-specific search strategies are provided elsewhere [7].

### Selection of Sources of Evidence

Titles, abstracts, and full texts were independently screened by 2 reviewers (out of AB, JH, and DB) using the Rayyan web application (Rayyan) [23]. Any discrepancies in the decisions were resolved by discussions within the review team.

### Data Charting Process and Data Items

A targeted and uniform extraction sheet was developed and consented to by the research team. We extracted study and design characteristics (year of publication, country where the study was conducted, objectives, number of participants or contacts) and assessed the technology-based counseling interventions by applying criteria from the Template for Intervention Description and Replication checklist [24] and from the revised Criteria for Reporting the Development and Evaluation of Complex Interventions guideline [25] to obtain a comprehensive overview of the interventions and their components. Data extraction on implementation success was guided by the conceptual framework for implementation outcomes, which comprises the implementation outcomes acceptability, adoption, appropriateness, feasibility, fidelity, implementation cost, penetration, and sustainability [17]. We adapted the definitions of the outcomes by specifying the intervention of interest and adjusting it to the research interest of our scoping review. Adaptations were consented to by the review team. The original and adapted definitions are listed in Table 1.

**Table 1:** Original and adapted definitions of implementation outcomes.

Outcome	Original definition according to Proctor et al [17]	Adapted definition for our review
Acceptability	“*Acceptability* is the perception among implementation stakeholders that a given treatment, service, practice, or innovation is agreeable, palatable, or satisfactory.”	*Acceptability* is the perception among implementation stakeholders of technology-based counseling that the intervention is agreeable, palatable, or satisfactory.
Adoption	“*Adoption* is defined as the intention, initial decision, or action to try or employ an innovation or evidence-based practice.”	*Adoption* is defined as the intention, initial decision, or action to try or employ a technology-based counseling intervention.
Appropriateness	“*Appropriateness* is the perceived fit, relevance, or compatibility of the innovation or evidence based practice for a given practice setting, provider, or consumer; and/or perceived fit of the innovation to address a particular issue or problem.”	*Appropriateness* is the perceived fit, relevance, or compatibility of the technology-based counseling intervention for the given practice setting, provider, and consumer; and/or perceived fit of the intervention to address a particular issue or problem.
Feasibility	“*Feasibility* is defined as the extent to which a new treatment, or an innovation, can be successfully used or carried out within a given agency or setting (Karsh 2004).”	*Feasibility* is defined as the extent to which the technology-based counselling intervention can be successfully used or carried out within a given agency or setting.
Fidelity	“*Fidelity* is defined as the degree to which an intervention was implemented as it was prescribed in the original protocol or as it was intended by the program developers (Dusenbury et al. 2003; Rabin et al. 2008).”	*Fidelity* is the degree to which a technology-based counseling intervention was implemented as it was prescribed in the original protocol or as it was intended by the program developers.
Implementation cost	“*Cost (incremental or implementation cost)* is defined as the cost impact of an implementation effort.”	*Implementation cost* is the cost impact of an implementation effort.
Penetration	“*Penetration* is defined as the integration of a practice within a service setting and its subsystems.”	*Penetration* is defined as the integration of a technology-based counseling intervention within a service setting.
Sustainability	“*Sustainability* is defined as the extent to which a newly implemented treatment is maintained or institutionalized within a service setting’s ongoing, stable operations.”	*Sustainability* is defined as the extent to which an implemented technology-based counseling intervention is maintained or institutionalized within an organization’s ongoing, stable operations.

Quotations from the included studies were extracted and assigned to the outcomes by 1 reviewer (out of DB or JW) and cross-checked for accuracy by another reviewer (out of DB or JW). Any discrepancies were resolved by consensus between these 2 reviewers.

### Synthesis of Results

Data on the implementation success of technology-based counseling interventions were summarized by applying the framework mentioned in the preceding section [17]. The following 8 conceptually distinct implementation outcomes were used as conceptual categories to operationalize *implementation success*: acceptability, adoption, appropriateness, feasibility, fidelity, implementation cost, penetration, and sustainability.

Our approach to data synthesis involved the following steps:

1. Concept specification: identification of dimensions in the definition of each conceptual category: as these conceptual categories incorporate various aspects [17] and therefore represent multidimensional concepts, a concept specification was performed by determining the dimensions of the categories [26,27]. Dimensions are defined as characteristics according to which empirical facts can be distinguished [28]. The 2 reviewers (DB and JW) independently identified dimensions matching the attributes of the conceptual categories [27] described by Proctor et al [17] with the characteristics of the extracted data. Consensus on the dimensions was reached through discussion between the 2 reviewers.
2. Reduction of data and assignment to dimensions in analysis matrices: the extracted data were reduced without paraphrasing and assigned to the dimensions using tables as analysis matrices.
3. Specification of the level of analysis: we specified the level at which data were provided (level of analysis: consumer, provider or providing institution, organization, setting, and administration), as indicated by Proctor et al [17].
4. Narratively synthesizing of findings and graphical presentation: findings were narratively synthesized and presented in the form of a net diagram.

Each synthesis step was cross-checked (DB and JW) and consent was obtained from the review team.

Study characteristics and characteristics of the included interventions are presented in narrative and tabular forms.

### Deviations From the Protocol

The prespecified method of conducting a Qualitative Comparative Analysis on the conditions of successful implementation of technology-based counseling interventions [7] could not be realized because of the heterogeneity of the data found in the literature. In addition, reports that were not written in English or German were excluded because of a lack of professional translation resources.

## Results

### Selection of Sources of Evidence

**Figure 1:**
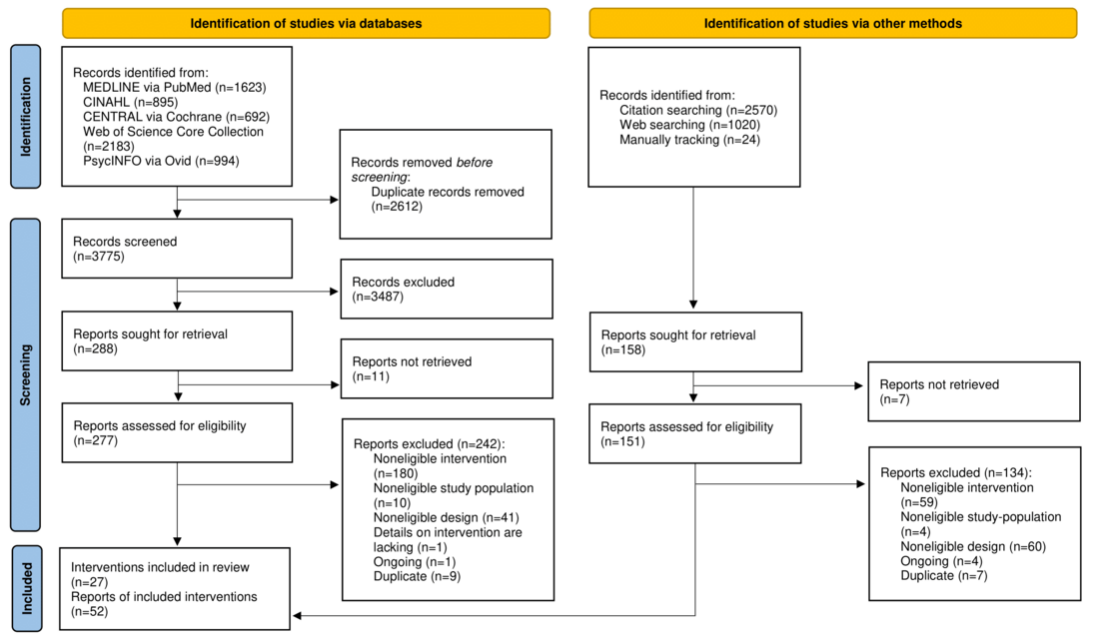
PRISMA (Preferred Reporting Items for Systematic Reviews and Meta-Analyses) flowchart of study selection.

The electronic database yielded 6387 records. After removing duplicates, we screened the titles and the abstracts of 3775 records, reviewed 277 full texts for eligibility, and included 35 records. We identified 3614 records from additional sources and assessed 151 full texts, of which 17 were included. Finally, 52 publications [29-80] reporting on 27 technology-based counseling interventions were included (Figure 1).

### Characteristics of Sources of Evidence

Interventions ranged from single counseling interventions, such as helpline services, to multicomponent programs combining nontechnology-based components, such as day care for people with dementia, with technology-based counseling for informal caregivers. All 27 interventions [29-80] focused on informal caregivers, mostly in combination with people with dementia, and 4 also addressed professional caregivers [29-32,34,35,37,40]. Counseling was provided by professionals from different disciplines, such as psychologists, geriatricians, or nurses. If volunteers were involved, they were professionally supervised or had received training. The technologies used for delivery included telephone, email, videoconferencing, social media, and chats, as well as web-based platforms. Additional (personalized) information material was frequently offered and delivered via email or postal mail. We differentiated the following types of interventions (refer to Table S1 in Multimedia Appendix 1): counseling via telephone or email and counseling via videoconferencing; web-based psychosocial intervention: information, communication, and counseling; videoconference- or telephone-based counseling combined with tele-monitoring or psychoeducation; and technology-based counseling as part of a comprehensive program with nontechnology-based components.

The design of the included studies varied (refer to Table S2 in Multimedia Appendix 1). Studies applying a quantitative descriptive design mostly focused on users’ demographics, topics discussed and advice provided in counseling sessions, and satisfaction with services. Few of the included studies exclusively focused on implementation, and we found process evaluation reports [79,80] related to 2 interventions. Furthermore, the publication type of the included reports varied greatly, as we aimed to depict the broad spectrum of interventions. In addition to research reports, we identified abstracts, letters to the editor, and practical project reports.

The interventions are displayed in Table S2 in Multimedia Appendix 1 and characteristics of the included studies are described in Table S3 in Multimedia Appendix 1.

### Results of Individual Sources of Evidence

Table 2 provides an overview of the data extracted from the included studies.

The label (“✓”) indicates the presence of data without any information on content or scope. None of the interventions included reported data in all categories. The information available ranged from 1 to 7 conceptual categories for each intervention (also refer to the analysis matrices in Multimedia Appendix 1).

**Table 2:** Overview of reported data on conceptual categories.

Intervention	References	Data extracted for conceptual categories							
		Acceptability	Adoption	Appropriateness	Feasibility	Fidelity	Implementation cost	Penetration	Sustainability
Admiral Nurse Helpline	[29-32]	✓	✓	✓	✓	✓		✓	✓
ADS^a^ Helpline	[33]	✓		✓				✓	✓
Alzheimer Helpline	[34,35]	✓		✓				✓	✓
Alz i-Connect	[36]	✓	✓	✓	✓		✓	✓	✓
CANDID^b^	[37]		✓	✓		✓		✓	✓
Care Consultation	[38]								✓
Care Consultation Plus	[38]	✓		✓					
Care Consultation/Care Consultation Plus^c^	[38]			✓		✓			
Coyne^d^ comparator	[39]								
Coyne^d^ experimental	[39]			✓	✓				
Coyne^c,d^	[39]			✓					
Helpline Alz Ass East Massa^e^	[40]	✓	✓	✓	✓	✓		✓	✓
Natale^d^	[41]			✓					
ODCC^f^	[42]		✓	✓					✓
Sabat^d^	[43]	✓		✓	✓				
Salfi^d^ nonanonym	[44-46]			✓					
Salfi^d^ anonym	[44-46]			✓					
Salfi^c,d^	[44-46]	✓		✓					
FITT-C^g^	[47-53]	✓	✓	✓	✓	✓			✓
FITT-D^h^	[54]	✓	✓	✓	✓	✓			
NVAMP^i^	[55]	✓		✓	✓				
ICSS^j^	[56-61]	✓		✓	✓				
InformCare	[62-64]	✓		✓	✓	✓	✓	✓	✓
Link2Care	[65]	✓	✓	✓				✓	✓
Online Coaching	[66]	✓		✓					✓
De Cola^d^	[67]	✓	✓	✓				✓	✓
Laver^d^	[68,69]	✓	✓	✓	✓	✓			
RCTM^k^	[70-73]			✓	✓				
Dementelcoach	[74-79]	✓	✓	✓	✓		✓	✓	
Nomura^d^	[80]	✓	✓	✓				✓	

^a^ADS: Alzheimer’s Disease Society.

^b^CANDID: Counseling and Diagnosis in Dementia.

^c^Assignment of the quotes to experimental and comparator intervention is not possible; we assume that the information is applicable for both interventions.

^d^When no name was reported, the name of the first author was assigned to the intervention.

^e^*Helpline Alz Ass East Massa*: Helpline of the Alzheimer's Association of Eastern Massachusetts.

^f^ODCC: Okayama Dementia Call Center.

^g^FITT-C: Family Intervention: Telephone Tracking – Caregiver.

^h^FITT-D: Family Intervention: Telephone Tracking – Dementia.

^i^NVAMP: Nurse Video With Assisted Modeling Program.

^j^ICSS: Internet-based Caregiver Support Service.

^k^RCTM: Residential Care Transition Module.

### Synthesis of Results

#### Acceptability

We defined *acceptability* as the perception among implementation stakeholders of technology-based counseling that the intervention is agreeable, palatable, or satisfactory [17]. Within the data related to the conceptual category (reported for 20 interventions [29-36,38,40,43-69,74-80]; refer to Table 2), we identified the dimensions *measures to promote acceptability* and *impact*, which can be further differentiated into *impact of parts of the service* and *impact of the overall service*. Measures to promote acceptability were reported, specifically from the perspective of the organizations. These were mainly aimed at promoting acceptability among providers, for example, through supervision, debriefings, or training. The impact on parts of the respective intervention or service was described from the perspective of both the provider and the consumer. The provider reported satisfaction with their role, whereas the consumer described individual parts of the service, including the helpfulness of the conversations, the competence of counselors, accessibility, materials, etc. The impact on the overall service was reported from the provider’s perspective, namely, satisfaction with the experience of the team members. Other interventions reported on the impact of the overall service from the consumer’s perspective, illustrated by relief, satisfaction with the intervention, or comfort with the service.

#### Adoption

*Adoption* is defined as the intention, decision, or action to use an intervention [17] (reported for 12 interventions [29-32,36,37,40,42,47-54,65,67-69,74-80]; refer to Table 2). Dimensions of adoption were *organizational motive*, *mode of decision*, and *uptake of interventions motivated internally or externally*. The motives of organizations included their commitment to helping families affected by dementia, connecting individuals to helpful information, providing access to support services, and providing support. Some authors described organizational motives in more general terms by referring to aspects that need to be addressed, for example, increased service demands or restrictions in the living conditions of people with dementia in the community. The mode of decision was characterized in different ways: as a response to developments in the setting, such as mobility restrictions or the increasing use of the internet in the target groups; as consent to participate at the organizational level; or as permission sought and obtained to implement the program. External reasons for the uptake of the specific intervention were evidence of the effectiveness of the intervention found in the literature or evidence indicating that the previously used mode of delivery needed to be adapted. Internally motivated uptake is based on the development, modification, or advancement of one’s own interventions.

#### Appropriateness

*Appropriateness* is understood as the perceived fit [17] of technology-based counseling for stakeholders, the setting, and the problems addressed. We defined the dimensions *overall compatibility with stakeholder needs*, *tailoring to individuals*, *skills and instruments for enhancing fit*, and *concepts for fit* for this conceptual category and found extensive information for all interventions [29-80]. If assignment of the quotes to experimental and comparator interventions was not possible, we assumed that the information was applicable for both interventions (refer to Table 2).

The dimension *overall compatibility with stakeholder needs* comprises information on accessibility, availability, tailoring to consumer groups, and usefulness of service. In the area of accessibility, the ways to access were described, ranging from the use of a single technology to multiple ways via email, telephone, and videoconferencing, or in combination with home visits. The availability of counseling in terms of service hours, for instance, permanently or during regular working hours on weekdays, was discussed against the background of availability when needed or in times of crisis. From the perspective of organizations, providers, and consumers, technology-based counseling was viewed as a mode of delivery that can solve logistical issues such as making appointments or long-distance travel. People who are homebound can access support without leaving their homes, and caregivers do not have to arrange substitute care. The limitations of remote delivery, such as the loss of context or consumers’ different capacities for using technology, were discussed from the perspective of the provider and the consumer. In addition, the advantages and disadvantages of anonymous services and services in which providers know the caregiver or the person with dementia are debated. Tailoring to consumer groups included services focusing on early-onset dementia or rare diseases, considering cultural and ethnic backgrounds, and providing multilingual services. To ensure the usefulness of the services, the appropriate amount and delivery of information were discussed. Tailoring to individuals included statements from the perspective of the provider that services were individualized, personalized, adapted, or flexible and aimed at responding to or addressing individual clients’ needs by offering the most appropriate intervention or the best approach to resolve problems. Providers’ skills for enhancing fit were described as specialist communication or counseling skills based on the training, knowledge, or experience of the person providing the service. Instruments used in organizations for enhancing fit comprise assessment instruments, information material, or written summaries of counseling sessions, such as letters, scripts, or proposals for individual use. Concepts for fit were reported from the providers’ perspective and described in terms of person-centered and holistic approaches by applying techniques such as validation or empathetic understanding and psychological strategies to enhance coping and problem-solving processes.

#### Feasibility

We defined *feasibility* as the extent to which technology-based counseling can be successfully used [17] and identified the dimensions *practicability*, *factors impeding feasibility*, and *factors promoting feasibility* (reported for 13 interventions [29-32,36,39,40,43,47-64,68-79]; refer to Table 2). The practicability of the interventions was stated from the perspective of the provider, the organization, or the setting. The quotes refer to the general practicability of the intervention, stating its successful application or conceptual clarity. Practicability was also discussed with reference to the use of technology. Factors impeding feasibility comprised general aspects such as lack of financial and staff resources, technology-related aspects such as legal standards and technical challenges, and the lack of visual and nonverbal cues when counseling was delivered via telephone. Special training of providers to overcome technical problems or to compensate for technology-related issues was reported as a factor promoting feasibility.

#### Fidelity

*Fidelity* is the degree to which an intervention was implemented as prescribed or intended [17] (reported for 9 interventions [29-32,37,38,40,47-54,62-64,68,69]; refer to Table 2). We determined *formalization of intervention* and *quality assurance in delivering the intervention* as dimensions of fidelity. *Formalization of intervention* was addressed by mentioning standardized manuals, guidelines, frameworks, protocols, or assessments. Proceedings for *quality assurance in delivering the intervention* comprised senior staff supervision to ensure adherence to the protocol and monitor fidelity, the analysis of audiotaped counseling sessions, and the use of adherence and competence scales.

#### Implementation Cost

On the basis of Proctor et al [17], we defined the conceptual category *implementation cost* as the cost impact of an implementation effort reported from the perspective of a provider or the providing institution (reported for 3 interventions [36,62-64,74-79]; refer to Table 2). We identified the dimensions *cost impact of delivery because of complexity of intervention*, *cost impact of implementation because of complexity of implementation strategy*, and *cost impact because of varying complexity of settings*. Data on the first dimension comprised the costs of delivering the complex intervention and its financing through previously paid travel costs. The costs of the implementation strategy were illustrated by the impact of existing and lacking financial resources for staffing on the implementation process. Within the third dimension, failed expansion or implementation because of a lack of resources was exemplified.

#### Penetration

Within the conceptual category of *penetration*, defined as a step of integrating the technology-based counseling intervention into the service setting ([17] reported for 11 interventions [29-37,40,62-65,67,74-80]; refer to Table 2), we identified 3 dimensions: *collaboration with stakeholders*, *access to the service*, and *spread*. Data from interventions describe cooperation with stakeholders to implement the intervention, initiated either by the implementing organization itself or jointly through cooperation with other stakeholders in the setting. Access to the service occurred through referrals from other stakeholders in the setting, for example, physicians, or through information from other sources, such as telephone books or newspapers. From the perspective of the implementing organization, the degree of utilization of the intervention by different professional groups (eg, physicians, social workers, and nurses) was described. The level of spread was reported from the perspective of the implementing organization as well as the setting and is often reflected as the regional spread of interventions, for example, at the national or international level.

#### Sustainability

Following Proctor et al [17] and consequently Steckler et al [81], we understand *sustainability* (reported for 13 interventions [29-38,40,42,47-53,62-67]; refer to Table 2) as the final phase of the diffusion process in which innovations become entrenched in organizations. We were able to identify specifications of the data in 3 dimensions: *routinization*, *passage*, and *incorporation*. These dimensions were mainly reported from the perspective of the implementing organization; once, the perspective of the administration was also taken. The data on the dimension *routinization* provided information on the permanence and the degree to which the intervention was established, especially related to the number of versions of the intervention developed, the stakeholders involved, or the period from the start of the program. The duration varied greatly, ranging from a recent introduction to a multiyear build-up with many contacts. Statements were found in the interventions indicating maintained procedures, with the (planned) transition to expand the intervention often explained from the perspective of the organization with the aim of maintaining the intervention. An administrative perspective was also taken, referring to the discontinuation of support after the end of the research project and, thus, the termination of the program. For example, this dimension was clarified by the integration of another target group or expansion to another region. Incorporation, for example, the final integration into (existing) organizational structures with the aim of maintaining the intervention, was mentioned, describing the dissemination of the service within existing structures.

### Graphical Presentation of Synthesized Data

**Figure 2:**
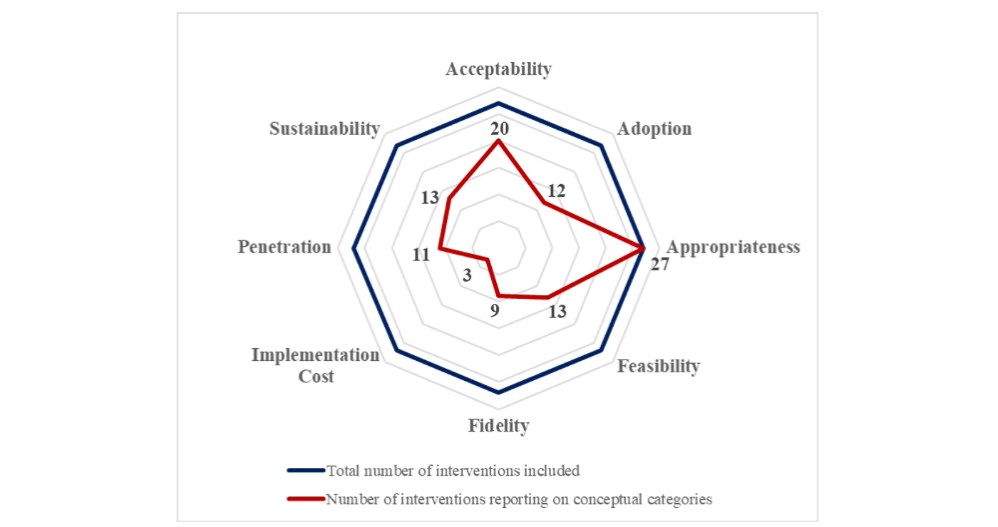
Net diagram on reported data of conceptual categories.

Figure 2 shows the graphical synthesis of the data. Data on the conceptual categories of *implementation success* were sought for 27 interventions (blue line). The number of interventions for which data were extracted is indicated by the red line. While the categories *appropriateness* and *acceptability* are largely covered, substantial parts of the other areas remain unconsidered (refer to Figure 2).

## Discussion

### Principal Findings

We aimed to review the knowledge about the implementation success of technology-based counseling interventions for people with dementia or their caregivers. In our scoping review, we included 52 publications that reported 27 interventions. Interventions were heterogeneous and ranged from single counseling interventions, such as helplines, to counseling as part of multicomponent programs. To operationalize *implementation success*, we used the 8 outcomes of the conceptual framework for implementation outcomes [17] as conceptual categories. Only a few studies evaluated the implementation. Reporting on implementation outcomes was found to be fragmentary, and the comprehensiveness of the information varied widely. Overall, the focus of reporting was on the outcomes of *appropriateness* and *acceptability*, which may be because great efforts were made to adapt the interventions to a vulnerable target population.

As our data show, reporting on *acceptability* is inconsistent in terms of the perspective taken: data reports on measures to promote acceptability, but only from the perspective of the provider. At the same time, reporting on impact from this perspective was underrepresented. As already discussed in the literature, there are difficulties in unifying the wealth of perspectives in the context of implementation research [16].

The information reported on the dimension of *adoption* illustrates the importance of the fit between organizational motives and the type of intervention chosen for successful implementation. Little data are available on the mode of decision but differences in organizational culture can be identified that may influence the success of implementation efforts. We found data indicating that decisions from administrative stakeholders had an impact on sustainability by limiting the duration of the implementation of an intervention. Increasing the administrators’ enthusiasm for implementing the intervention by promoting familiarity with the specific intervention and using the effect of name-brand recognition may facilitate long-term commitment [82]. In some cases, the uptake of a specific intervention was based on evidence of its effectiveness, and there is a need for further effectiveness trials to expand the evidence base for decision-making regarding the implementation of technology-based counseling interventions.

The data provided on *appropriateness* comprised the largest amount of information extracted for categories and document the efforts undertaken to fit the target population. In addition to general measures to enhance the perceived fit of individualized support services, providers’ skills and instruments, as well as concepts applied by individual providers, were described. Tailoring and personalizing counseling services to individuals’ needs has been associated with the usefulness of information and support [55-65]. The benefits and limitations of using technology for delivering counseling are discussed against the background of statements by consumers who would have preferred a different mode of communication with counselors [36]. On the basis of participants’ attrition, González-Fraile et al [9] reported that remote support or training interventions appear to be less acceptable to informal caregivers of people with dementia than control interventions, which may limit their applicability in community settings. Further research is needed to determine whether services that are accessible both face to face and technology-based can provide appropriate accessibility and improve the perceived fit of the target populations.

Information on *feasibility* comprised the successful implementation of interventions. Although factors impeding feasibility, such as legal issues and technical challenges, were reported, we found no information on failed or unsuccessful implementation. Barriers to the implementation of eHealth interventions described in the literature are, among others, the lack of digital literacy in the target population and staff’s uncertainties and insecurities about their coaching competences [83]. According to Proctor et al [17], the concept of *feasibility* is typically “invoked retrospectively as a potential explanation of an initiative’s success or failure.” Thus, a more comprehensive reporting of factors promoting or impeding feasibility may inform the implementation of interventions in future projects and may contribute to increasing the implementation success of technology-based counseling interventions.

Ways of formalizing the interventions to ensure fidelity in the delivery of interventions were mentioned, but manuals or guidelines were not made accessible along with publications. In addition, measurements to ensure fidelity were described for some interventions, but the results of assessments, as well as details on aspects where deviations occurred, were not reported. After critically reviewing the literature on the use of fidelity implementation frameworks in early intervention, Lemire et al [84] also stated gaps in defining and assessing implementation fidelity. Drawing on preexisting conceptualizations, the authors proposed a definition of fidelity that comprises the 4 components: adherence, exposure, quality, and participant responsiveness [84].

The cost impact of implementation efforts was rarely reported for the included interventions. Factors that influence the costs are the complexity of the specific intervention, the strategy used for implementation, and the delivery setting [17]. Despite the costs incurred in setting up the technical infrastructure, the costs for remote delivery were lower than when counseling was provided face to face [36,68,69]. Owing to the lower costs, eHealth interventions are considered suitable for widespread implementation [16]. The provision of information on implementation costs is essential to compare the cost impact of different interventions and to inform decisions regarding the uptake of a specific intervention [17].

The data reported on *penetration* often indicates access to the service in multiple ways, which seems to match the preferences and capabilities of consumers. As reported by Jelly et al [85], caregivers use dementia support services primarily when services are able to meet consumers’ individual needs. However, it is important to keep in mind that, from an organizational point of view, these extended access options need to be served simultaneously. In particular, cooperation with other stakeholders seems to be central to integrating counseling services into a service setting, but this was only highlighted by some authors in the included publications.

The sustainable anchoring of diffusion processes is described as a difficult phase in the implementation process of support services for caregivers of people with dementia. The reasons for this include a lack of understanding of the barriers to sustainable implementation in practice and a lack of long-term funding [86]. The problem is substantiated in that, as long as researchers focus on measuring the effectiveness of the interventions, the potential goal is not fully realized. However, there are models that can support this sustainable implementation [87].

There are several theoretical approaches, such as generalized theories, models, or frameworks, that address different aspects of implementation [88]. Frameworks “describe more loosely structured constellations of theoretical constructs... or prescriptive approaches for accomplishing implementation goals” [88]. By providing clarity in terms and definitions [88], frameworks contribute to shared language in implementation research. There are different types of frameworks focusing on processes or determinants or evaluations [88]. Evaluation frameworks, such as the conceptual framework for implementation outcomes introduced by Proctor et al [17] and the Reach, Efficacy, Adoption, Implementation, Maintenance (RE-AIM) planning and evaluation framework [89,90], offer guidance on identifying results that can be used to evaluate implementation efforts [88]. While the RE-AIM framework describes outcomes across 5 domains (reach, effectiveness, adoption, implementation, and maintenance) [90], Proctor et al [17] present the concept of 8 implementation outcomes, which are differentiated from service system and treatment outcomes. Implementation outcomes are defined as “the effects of deliberate and purposive actions to implement new treatments, practices, and services” and are reported from different levels of analysis (eg, the provider or the consumer perspective) [17]. Serving as conceptual categories of the implementation success [17], these outcomes provided the appropriate approach to operationalize the object of interest—the implementation success of technology-based counseling interventions in dementia—in our review.

To increase the clarity of terminology used in implementation research, Proctor et al [17] proposed the definitions of 8 conceptually distinct implementation outcomes as a “working taxonomy,” including different aspects of implementation success and thus creating a comprehensible framework.

The use of outcomes as conceptual categories was sometimes challenging in our case. The mapping of the extracted data, in particular, was sometimes difficult because of the conceptual similarity of some outcomes, for example, penetration and sustainability, and the inconsistent use of terminology found in the literature. When determining the levels of analysis, we sometimes included additional perspectives, as described by Proctor et al [17].

Altogether, the lack of process evaluation studies, the fragmented reporting, and the unclear use of terms and concepts made it impossible to determine the extent of implementation success of technology-based counseling interventions in dementia care. Because of the impaired comparability of data, we were not able to assess how the different types of interventions affect the conceptual categories of acceptability, adoption, appropriateness, feasibility, fidelity, implementation cost, penetration, and sustainability. For instance, we found data on *appropriateness* for all interventions, but the consumer-perceived usefulness of services was referred to for only 5 interventions. These 5 interventions comprised helplines that provided counseling via telephone [29-32,55] and web-based psychosocial interventions that provided information, communication, and counseling [56-65]. The reported information does not allow any conclusions to be drawn on how the types or components of interventions have an impact on implementation success. Gaining further insight into this issue is important for developing future interventions that can be implemented successfully.

Adherence to the framework for developing and evaluating complex interventions [6] may help overcome these problems, as proper process evaluation and exploration of conditions for implementation are recommended. The update to the Medical Research Council guidance states that “[e]arly consideration of implementation increased the potential of developing an intervention that can be widely adopted and maintained in real-world settings” [6] and thus can increase the success of implementation efforts. In addition, the conceptual clarity of the terms and concepts used in implementation research is needed to enhance transparency. This can be achieved by applying theoretical approaches that are “encapsulated as generalized theories, models, or frameworks” [88]. The consistent use of terms not only creates clarity but also forms the basis for better reporting on the success of implementation efforts, as Lengnick-Hall et al [91] proposed as the first of 6 practical recommendations for improved implementation outcomes reporting.

### Strengths and Limitations

We followed a theory-driven approach to review the available evidence on implementation success. As we examined a broad topic with evidence emerging from studies in various designs, a scoping review proved appropriate. We performed a comprehensive and methodologically rigorous systematic literature search and included a variety of technology-based counseling interventions for people with dementia and their informal caregivers. Differentiating counseling from interventions focusing on education and information or from psychotherapeutic approaches brought challenges that we overcame through intensive discussions in the review team. Although we were able to include a considerable number of publications, it was not possible to make reliable statements about the implementation success of technology-based counseling interventions in dementia because of the inconsistent database as well as the heterogeneity in terminology and concepts.

### Conclusions

We applied 8 conceptually distinct categories to operationalize the implementation success of technology-based counseling interventions for people with dementia and their informal caregivers. We found considerable data for the categories *appropriateness* and *acceptability*, and limited data on *sustainability*, *feasibility*, *adoption*, *penetration*, *fidelity*, and *implementation cost*. There is an imbalance in the scope and depth of the reported data on the conceptual categories, and the data extracted from the included publications only partially covered the concept of *implementation success*.

This highlights the need for a systematic evaluation accompanying the implementation of technology-based counseling interventions in the context of dementia. Adherence to guidelines for the development and evaluation of interventions and to guidelines or recommendations for reporting conceptualizations, measurements, and results on implementation outcomes is needed to expand knowledge on the effectiveness of implementation efforts and may foster the implementation of complex interventions in diverse contexts.

## Appendix

Multimedia Appendix 1 Description of included intervention programs, description of studies reporting on included intervention programs, and analysis matrices.

Multimedia Appendix 2 Preferred Reporting Items for Systematic reviews and Meta-Analyses extension for Scoping Reviews (PRISMA-ScR) Checklist.

## Abbreviations

PRISMA-ScR: Preferred Reporting Items for Systematic Reviews and Meta-Analyses Extension for Scoping Reviews
RE-AIM: Reach, Efficacy, Adoption, Implementation, Maintenance
