# Supplementary material for: The Implementation Success of Technology-Based Counseling in Dementia Care: Scoping Review
Source: JMIR Aging. 2024 Jan 25;7:e51544. doi: 10.2196/51544 (PMC10853855; doi:10.2196/51544)
Supplement: Multimedia Appendix 1 [file aging_v7i1e51544_app1.docx]

Multimedia Appendix

The Implementation Success of Technology-Based Counseling in Dementia Care: Scoping Review

**Content**

[Chapter A: Description of included intervention programs and studies 2](#_Toc155857424)

[Table S1: Description of included intervention programs 2](#_Toc155857425)

[Table S2: Description of studies reporting on included intervention programs 6](#_Toc155857426)

[Chapter B: Analysis matrices for implementation outcomes 10](#_Toc155857427)

[Table S3: Analysis matrix for ‘acceptability’ 10](#_Toc155857428)

[Table S4: Analysis matrix for ‘adoption’ 16](#_Toc155857429)

[Table S5: Analysis matrix for ‘appropriateness’ 21](#_Toc155857430)

[Table S6: Analysis matrix for ‘feasibility’ 49](#_Toc155857431)

[Table S7: Analysis matrix for ‘fidelity’ 53](#_Toc155857432)

[Table S8: Analysis matrix for ‘implementation cost’ 55](#_Toc155857433)

[Table S9: Analysis matrix for ‘penetration’ 56](#_Toc155857434)

[Table S10: Analysis matrix for ‘sustainability’ 60](#_Toc155857435)

# Chapter A: Description of included intervention programs and studies

## Table S1: Description of included intervention programs

| **Intervention^a^**  **(References)** | **Type of intervention (technology used)** | **Objective(s)** | **Duration/**  **Frequency/**  **Period** | **Provider** | **Consumer** |
| --- | --- | --- | --- | --- | --- |
| Admiral Nurse Helpline  [29–32] | Counselling via telephone (follow-up information by email or by post) | Providing specialist support as informants, educators and role models;  Helping families to cope with dementia | calls often > 30 min  N/A  N/A | Trained nurses with expertise in dementia care | Informal and professional carers;  People with dementia |
| ADS  [33] | Counselling via telephone | Providing support, advice and information | x̅ = 6.18 min  N/A  N/A | N/A | Informal carers;  People with dementia |
| Alzheimer Helpline  [34,35] | Counselling via telephone and email | Counselling, providing information, referral | x̅ = 11-15 min  N/A  N/A | Social workers, psychologists, nurses | Informal and professional carers;  People with dementia |
| ALZ i-Connect  [36] | Counselling via videoconferencing | Providing the patient and family a welcoming and concerned person to introduce them to the services and supports available, connection of families;  Giving information | N/A  N/A  N/A | Helpline staff and care consultants with a bachelor’s or master’s degree in social work, counselling, or related degree | People with dementia and their families |
| CANDID  [37] | Counselling via telephone (alternative: also via email, in person or by post) | Providing a point of contact and information for patients and carers;  Providing clinical management advice and guidance to GPs | N/A  N/A  N/A | Trained nurses/counsellors  (reviewed by a consultant neurologist and a psychiatrist) | People with dementia;  Informal and professional carers |
| Care Consultation  [38] | Counselling via telephone | Providing emotional support, information and referrals to additional resources in the local community | N/A  N/A  N/A | Care consultant with master’s degree in social work, counselling or related field | Informal carers |
| Care Consultation Plus  [38] | Counselling via telephone | Providing emotional support, information and referrals to additional resources in the local community | N/A  Initial + follow up call  1 month^d^ | Care consultant with master’s degree in social work, counselling or related field | Informal carers |
| Coyne^a^ comparator  [39] | Counselling via telephone | Providing information, referral, education, and counselling | N/A  once  N/A | Staff with extensive experience working with people with dementia and informal carers | Informal carers |
| Coyne^a^ experimental  [39] | Counselling via telephone | Providing information, referral, education, and counselling | N/A  Helpline call + biweekly follow-up calls  8 weeks | Staff with extensive experience working with people with dementia and informal carers | Informal carers |
| Helpline Alz Ass East Massa  [40] | Counselling via telephone | Providing access to comprehensive services;  Providing support to carers | x̅ = 14 min  N/A  N/A | Psychologist, trained volunteers | Informal and professional carers |
| Natale^a^  [41] | Counselling via telephone | Improving disease management | N/A  monthly  6 months | Neuropsychologist, trained geriatrician | Informal carers |
| ODCC  [42] | Counselling via telephone | Listening to the problems of clients and carers;  Providing with useful information;  Advising on necessary medical consultations | x̅ = 20.2 min  N/A  N/A | Experienced experts (family or public carers) | People with dementia;  Informal carers |
| Sabat^a^  [43] | Counselling via email | Providing education, counselling, and psychosocial support | N/A  N/A  approx. 3 years | Psychologist | Informal carer |
| Salfi^a^ nonanonym  [44–46] | Technology-based counselling as a part of a comprehensive program with non-technology-based components | Providing telephone support as follow-up to their other support programs, carer- or provider-initiated | N/A  N/A  N/A | Educational qualifications in nursing or gerontology | Informal carers |
| Salfi^a^ anonym  [44–46] | Counselling via telephone | Providing confidential and carer-initiated telephone support | N/A  N/A  N/A | Health care professionals | Informal carers |
| FITT-C  [47–53] | Counselling via telephone | Providing information about dementia, recommendations for resources, and emotional support to reduce depres-  sion and burden in dementia carers | Initial 60 min, follow-up 15–30 min  16 telephone contacts  6 months | Trained master’s level therapists (mental health counsellors, social workers, nurses) | Informal carers |
| FITT-D  [54] | Counselling via telephone | Providing emotional support, directing carers to appropriate resources, encouraging carers to attend to their own physical, emotional and social needs, and teaching carers strategies to cope with ongoing problems | Initial 60 min, follow-up 15–30 min  23 telephone contacts  12 months | Trained master’s level therapists | Informal carers |
| NVAMP  [55] | Counselling via telephone | Providing support for carers | x̅ = 18.3 min  N/A  N/A | Trained master’s level gerontological clinical nurse specialists | Informal carers |
| ICSS  [56–61] | Web-based psychosocial intervention: information, communication and counselling | Helping carers manage more effectively the burden of caring for a family member with dementia  Improvement the overall quality of life for the carer and the care recipient | N/A  N/A  6 months | Experienced clinicians with a clinical background in occupational therapy or social work | Informal carers |
| InformCare  [62–64] | Web-based psychosocial intervention: information, communication and counselling | Offering information resources and interactive services to enable professional and peer support | N/A  N/A  N/A | Psychologist or social workers | Informal carers |
| Link2Care  [65] | Web-based psychosocial intervention: information, communication and counselling | Increase carers well-being and coping skills through convenient access to information, connection to other carers, and other services | N/A  N/A  N/A | Technical experts in caregiving, law, and health | Informal carers |
| Online Coaching Program  [66] | Web-based psychosocial intervention: information, communication and counselling | Providing individualized support and information to carers | N/A  N/A  N/A | Social worker | Informal carers |
| De Cola^a^  [67] | Videoconference- or telephone-based counselling combined with tele-monitoring or psychoeducation | Providing a health care service for the elderly and support to their carers | x̅ = 30 min  Weekly  N/A | Neurologist, psychologist  (assistance by other health care professionals) | People with dementia;  Informal carers |
| Laver^a^  [68,69] | Videoconference- or telephone-based counselling combined with tele-monitoring or psychoeducation | Improving problem-solving skills, education, and building skills;  Addressing stress management;  Working with the dyad to enhance activity engagement in the person with dementia | x̅ = 60 min  8 sessions (incl. 2 home visits)  16 weeks | Trained occupational therapists | Informal carers;  People with dementia |
| RCTM  [70–73] | Videoconference- or telephone-based counselling combined with tele-monitoring or psychoeducation | Providing support and skills-building to help carers adapt to their care recipient’s transition to longterm care | x̅ = 81.3 min  6 sessions  4 months  + optional ad hoc sessions over 12 months | Trained transition counsellors | Informal carers |
| Dementelcoach  [74–79] | Technology-based counselling as part of a comprehensive program with non-technology-based components | Providing emotional, social, and practical support for carers;  Increasing the support experience by carers and enhance their ability to cope with the consequences of dementia | x̅ = 30-45 min  8-10 calls  20 weeks | Trained professional carers | Informal carers |
| Nomura^a^  [80] | Technology-based counselling as part of a comprehensive program with non-technology-based components | Empowerment of people with dementia and their carers;  Coaching of problem-focused coping | N/A  monthly  N/A | Trained social worker or public health nurses (certified care managers) | Informal carers;  People with dementia |

*Abbreviations*: ADS: Alzheimer's Disease Society; CANDID: Counselling and Diagnosis in Dementia; FITT-C: Family Intervention: Telephone Tracking – Caregiver; FITT-D: Family Intervention: Telephone Tracking – Dementia; Helpline Alz Ass East Massa: Helpline of the Alzheimer's Association of Eastern Massachusetts; ICSS: Internet-based Caregiver Support Service; min: minutes; N/A: not applicable/not available; NVAMP: Nurse Video With Assisted Modeling Program; ODCC: Okayama Dementia Call Center; RCT: Randomized controlled trial; RCTM: Residential Care Transition Module.

*Notes*^: a^When no name is reported, the name of the first author was assigned to the intervention.

^d^An additional booster call was made within a month; the exact time was not specified.

## Table S2: Description of studies reporting on included intervention programs

| **Intervention^a^** | **Reports included^c^**  **Country** | **Study design** | **Aim(s) of report** | **Number of participants / contacts** |
| --- | --- | --- | --- | --- |
|  |  |  |  |  |
| Admiral Nurse Helpline | **Wilkinson 2016** [29]  UK | Quantitative descriptive and qualitative study | Analysis and evaluation of the intervention | n=305 contacts |
|  | Brown 2020a [30]  UK | N/A (practical report) | Exploring the use of telephone helplines during SARS-CoV-2 pandemic  Reflection on the experience of a dementia specialist helpline nurse | N/A |
|  | **Brown 2020b** [31]  UK | Case study | Presentation and reflection of a call to the Admiral Nurse Dementia Helpline | n=1 participant |
|  | **Drayton 2020** [32]  UK | Case study | Discussion of the role of managing complexity on a dementia telephone helpline | n=1 participant |
| ADS Helpline | Gilliard 1998 [33]  UK | Quantitative descriptive (letter to editor) | Brief description of project results | n=60 contacts |
| Alzheimer Helpline | **Jansen 2007** [34]  Germany | Quantitative descriptive | Evaluation of helpline service (e.g., caller demographics, situation of people with dementia, ways of access, topics discussed, satisfaction with service) | n=4,837 contacts |
|  | **Pendergrass 2019** [35]  Germany | Quantitative descriptive | Evaluation of helpline service (e.g., caller demographics, topics discussed, ways of access) | n=3,744 participants |
| ALZ i-Connect | Tousi 2017 [36]  USA | Quantitative descriptive | Evaluation of a novel implemented audiovisual care consultation | n=11 participants |
| CANDID | Harvey 1998 [37]  UK | Quantitative descriptive | Audit and evaluation of the introduction of a novel support service | n=1,121 contacts |
| Care Consultation / Care Consultation Plus^b^ | Hodgson 2021 [38]  USA | RCT | Evaluation of the effectiveness of Helpline “Care Consultation” and “Care Consultation Plus” conditions | n=445 participants |
| Coyne^a^ comparator / experimental^b^ | Coyne 1995 [39]  USA | RCT | Evaluation of the effectiveness of standard and extended helpline services | n=62 participants |
| Helpline Alz Ass East Massa | Silverstein 1993 [40]  USA | Quantitative descriptive | Evaluation of helpline services (e.g., caller demographics, demographics of people with dementia, ways to access, reasons for calling, information provided, satisfaction with service) | n=100 participants |
| Natale^a^ | Natale 2012 [41]  Italy | Non-randomized trial (letter to editor) | Evaluation of the impact on disease-management effectiveness | n=52 participants |
| ODCC | Nakano 2018 [42]  Japan | Quantitative descriptive | Evaluation of a telephone support system (e.g., callers demographics, demographics of people with dementia, topics discussed, advice provided) | n=1,485 contacts |
| Sabat^a^ | Sabat 2011 [43]  USA | Case study | Examination of the effect of email education, counselling, and psychosocial support on the carer’s evolving subjective experience | n=1 participant (1,276 contacts) |
| Salfi^a^ nonanonym / anonym^b^ | **Salfi 2004** [44]  Canada | Case study (dissertation) | Exploring the intervention of telephone support | n=2 agencies providing telephone support (n=8 callers and n = 4 provider participated) |
|  | **Salfi 2005** [45]  Canada | Qualitative study |  |  |
|  | Spilsbury 2006 [46]  Canada | Comment on Salfi 2005 | Reflection and discussion of study methodology and results |  |
| FITT-C | National Institutes of Health 2008 [47]  USA | N/A (registry entry) | N/A | N/A |
|  | Tremont 2011 [48]  USA | RCT (conference abstract) | Exploration whether carer characteristics were related to credibility and expectancy  Examination whether these variables were related to carers’ early response to two interventions | n=133 participants |
|  | Tremont 2013a [49]  USA | RCT (conference abstract) | Examination of the efficacy of FITT-C for reducing distress | n=237 participants |
|  | **Tremont 2013b** [50]  USA | RCT (study protocol and baseline data) | Description of study design, methodology, and baseline data | n=250 participants |
|  | Tremont 2014 [51]  USA | RCT (conference abstract) | Examination of the effects of FITT-C on community support and healthcare use | n=250 participants |
|  | **Tremont 2015** [52]  USA | RCT | Examination of the effects of a telephone-based intervention on carer well-being | n=250 participants |
|  | **Tremont 2017** [53]  USA | RCT | Examination of the effects of a telephone-delivered intervention on community support and healthcare use | n=250 participants |
| FITT-D | Tremont 2008 [54]  USA | RCT | Examination of the preliminary effectiveness of FITT-D for reducing carer burden, depression, and reactions to memory and behaviour problems | n=60 participants |
| NVAMP | Chang 2004 [55]  USA | Qualitative study | Description of carers’ perceived reasons for helpfulness of telephone calls | n=83 participants |
| ICSS | Chui 2005 [56]  Canada | Qualitative study (conference abstract) | Reporting on the developmental phase of a new internet-based support services | n=3 participants |
|  | **Chui 2008** [57]  Canada | Mixed methods study (dissertation) | Description and explanation (non-)usage behaviour of e-health services among carers | n=46 participants |
|  | **Chui 2009** [58]  Canada | Mixed methods study | Assessment of the usability of a new Internet-based Caregiver Support Service  Evaluation of the effects on health outcomes of carers | n=28 participants |
|  | Chui 2010a [59]  Canada | Mixed methods study | Explore the usage behaviour of users of e-health services  Exploration of factors associated with uptake and use of an internet-mediated intervention for carers | n=46 participants |
|  | Chui 2010b [60]  Canada | Qualitative study | Analysis of the content and process of the new intervention  Explore how the client-centred concepts can be applied to Internet-mediated intervention | n=28 participants |
|  | Chui 2011 [61]  Canada | Qualitative study | Improvement of understanding of family carers’ use of Web-based intervention support | n=14 participants |
| InformCare | **Barbabella 2016** [62]  Italy, Sweden, Germany | Mixed methods study | Examination of the impact of the Web-based psychosocial intervention on carers | n=123 participants |
|  | Lamura 2017 [63]  Italy, Sweden, Germany | N/A (project report) | Description of the platform content, the piloting and implementation process, critical reflections and sustainability | N/A |
|  | **Barbabella 2018** [64]  Italy, Sweden, Germany | Mixed methods study | Report on the usage and usability evaluation of a multicomponent Web-based program for psychosocial support for informal carers | n=123 participants |
| Link2Care | Kelly 2003 [65]  USA | Quantitative descriptive | Report on users’ demographics and satisfaction | N/A |
| Online Coaching | Rentz 2010 [66]  USA | Quantitative descriptive | Report on utilization rates and users’ satisfaction | n=121 participants |
| De Cola^a^ | De Cola 2016 [67]  Italy | Quantitative descriptive | Evaluation of usability and effects of the telehealth system on carers and people with dementia | n=38 participants |
| Laver^a^ | Australian New Zealand Clinical Trials Registry 2017 [68]  Australia | N/A (registry entry) | N/A | N/A |
|  | **Laver 2020** [69]  Australia | RCT | Determination whether delivery of a dyadic intervention using telehealth was noninferior to delivery of the same program using home visits | n=63 participants |
| RCTM | National Institutes of Health 2016 [70]  USA | N/A (registry entry) | N/A | N/A |
|  | Gaugler 2020 [71]  USA | N/A (study protocol) | N/A | N/A |
|  | **Statz 2021** [72]  USA | Mixed methods study | Identification of areas of guilt experienced by carers of people with dementia after long-term care transition  Exploration of carers’ characteristics predicting heightened feelings of guilt  Identify how a counselling intervention can provide practical support to help alleviate carers’ guilt following transition | n=83 participants |
|  | **Zmora 2021** [73]  USA | Mixed methods study | Improvement of understanding of the interpersonal and contextual factors that influence carer-staff relationships in residential long-term care facilities  Identify targets for future interventions to improve relationships | n=85 participants |
| Dementelcoach | van Mierlo 2011 [74]  The Netherlands | Non-randomized trial (conference abstract) | Evaluation of the impact of the intervention on informal carers and trained telecoaches  Investigation of the effect of the intervention on the professional carer’s work satisfaction, work experience and self-esteem | N/A |
|  | **van Mierlo 2012** [75]  The Netherlands | Non-randomized trial | Evaluation of the effectiveness of telephone coaching on burden and mental health problems of informal carers | n=54 participants |
|  | Netherlands Trial Register 2015 [76]  The Netherlands | N/A (registy entry) | N/A | N/A |
|  | Droes 2019a [77]  The Netherlands | RCT (conference abstract) | Evaluation of the feasibility, implementation and (cost-) effectiveness of the individualized Meeting Centers Support Program (iMCSP) | N/A |
|  | **Droes 2019b** [78]  The Netherlands | RCT | Exploration of the effectiveness of the individualized Meeting Centers Support Program (iMCSP) consisting of DemenTalent, Dementelcoach (telephone coaching), and STAR e-Learning for carers, compared to regular MCSP and No day care support. | N/A |
|  | **Van Rijn 2020** [79]  The Netherlands | Process evaluation | Identification of facilitating and impeding factors of linking Dementelcoach and STAR e-Learning to existing Meeting Centres for people with dementia and their informal carers | n=15 participants |
| Nomura^a^ | Nomura 2009 [80]  Japan | Process evaluation | Description of the implementation and process evaluation of the intervention | n=68 participants |

*Abbreviations*: ADS: Alzheimer's Disease Society; CANDID: Counselling and Diagnosis in Dementia; FITT-C: Family Intervention: Telephone Tracking – Caregiver; FITT-D: Family Intervention: Telephone Tracking – Dementia; Helpline Alz Ass East Massa: Helpline of the Alzheimer's Association of Eastern Massachusetts; ICSS: Internet-based Caregiver Support Service; N/A: not applicable/not available; NVAMP: Nurse Video With Assisted Modeling Program; ODCC: Okayama Dementia Call Center; RCT: Randomized controlled trial; RCTM: Residential Care Transition Module.

*Notes*: ^a^When no name is reported, the name of the first author was assigned to the intervention.

^b^Assignment of the quote(s) to experimental and comparator intervention not possible, we assume, the information is applicable for both interventions.

^c^If several publications on an intervention have been identified through the systematic literature search, main publications are marked in bold type, other
reports are considered as related publications.

# Chapter B: Analysis matrices for implementation outcomes

## Table S3: Analysis matrix for ‘acceptability’

| **Original definition of ‘acceptability’ according to Proctor et al. [17]:**  “*Acceptability* is the perception among implementation stakeholders that a given treatment, service, practice, or innovation is agreeable, palatable, or satisfactory.” | | **Adaptation of the definition:**  *Acceptability* is the perception among implementation stakeholders of technology-based counselling that the intervention is agreeable, palatable, or satisfactory. | | | |
| --- | --- | --- | --- | --- | --- |
| **Intervention^a^** | **Quote** | | **Dimensions of ‘acceptability’ (level of analysis)** | | |
|  |  |  | **Measures to promote acceptability** | **Impact** | |
|  |  |  |  | **Of parts of the service** | **Of the overall service** |
| Admiral Nurse Dementia Helpline [29–32] | Clinical supervision and continuing professional development (CPD) are essential for Admiral Nurses to provide their specialist role. All nurses have access to regular clinical supervision to reflect on and discuss calls. During the pandemic, additional peer support and debriefs at the end of each shift were established to better manage the complex nature of calls at this time. This post-shift support was in addition to the usual daily support from a shift coordinator. | | Clinical supervision and continuing professional development are essential (Org.)  have access to regular clinical supervision (Org.)  additional peer support and debriefs (Org.) |  |  |
|  | John contacted the helpline again the following week and the same nurse who had spoken to him previously was able to call him back. He explained that the meeting with the multidisciplinary team had gone well and attributed this to the confidence and knowledge he had gained by contacting the helpline. John felt empowered and better equipped to manage the situation as a result of the support and information provided by the nurse. | |  |  | confidence and knowledge he had gained by contacting the helpline (Con.)  felt empowered and better equipped to manage the situations (Con.) |
|  | Furthermore, the nurses running the helpline often work in isolation. Therefore, because the nature of their work, there is a need for helpline nurses to have time to reflect on their practice and access to clinical supervision. | | Need (…) to have time to reflect on their practice and access to clinical supervision (Org.) |  |  |
|  | Whilst it is difficult to identify specific and measurable outcomes Michael stated that he felt he had been supported, and that he felt he had a better understanding of how to help his father and was relieved to have a tangible plan to get more help for his parents at home. | |  |  | he had been supported (Con.)  a better understanding of how to help (Con.)  relieved to have a tangible plan (Con.) |
|  | The implications of displaying such a high degree of empathy, with such underpin caller satisfaction with the service | |  |  | caller satisfaction (Con.) |
|  | The vast majority of calls (N=277; 91%) included thanks from the caller, and often also praise for, and appreciation of, the helpline service. Three callers also offered a donation to Dementia UK. Callers typically offered more – sometimes much more – than a basic “thank you” for the call-taker’s help. For example, they said: “I’m so grateful”; “I really appreciate that”; “I think you’re brilliant”; “thanks ever so much”; “Thank you for all your valued support”; “Thanks very much indeed for all your help”; “Thank you so much for that”. Sometimes the praise and thanks offered was even more extensive and/or effusive. | |  |  | thanks from the caller (Con.)  praise for, and appreciation of the helpline service (Con.) |
| ADS helpline [33] | Concerning the telephone helpline staff themselves, most had undergone a training day on receiving calls from people with dementia and said they felt positive after the call. This feeling often correlated with an ability to do something practical, like sending some literature or referring back to the GP. Helpline staff sometimes felt less certain about the value of their role, especially when, as they put it, `they had simply listened'. | | undergone a training day (Org.) | felt positive after the call (…) correlated with an ability to so something practical (Prov.)  felt less certain about the value of their role (Prov.) |  |
| Alzheimer helpline [34,35] | Die Bewertung der Beratung und Information am Alzheimer-Telefon konnte in allen Jahren nur anhand jeweils einer Stichprobe von maximal 22 Fragebögen erfolgen. Der überwiegende Teil der Anrufer war mit dem Angebot zufrieden, empfand das Gespräch als hilfreich und die Beraterin als kompetent und freundlich. Auch konnte alle dem Satz zustimmen, dass sich die Beraterin genügend Zeit genommen hat. Die meisten der Befragten fanden die Telefonzeiten und die Erreichbarkeit befriedigend, aber ähnlich wie in den letzten Jahren wurde von einigen der Anrufer eine Ausweitung der Telefonzeiten gewünscht. | |  | empfand das Gespräch als hilfreich (Con.)  Beraterin als kompetent und freundlich (Con.)  genügend Zeit genommen (Con.)  Telefonzeiten und die Erreichbarkeit befriedigend (Con.)  Ausweitung der Telefonzeiten gewünscht (Con.) | war mit dem Angebot zufrieden (Con.) |
|  | *The evaluation of the counselling and information provided by the Alzheimer helpline was based on a sample of a maximum of 22 questionnaires in all those years. The majority of the callers were satisfied with the service, found the conversation helpful and the counsellor competent and friendly. All of them also agreed that the counsellor took enough time. Most of the respondents found the telephone hours and the accessibility satisfactory, but similar to the last years, some of the callers wished for an extension of the telephone hours.*  *(translated by authors)* | |  | *found the conversation helpful (Con.)*  *the counsellor competent and friendly (Con.)*  *telephone hours and the accessibility satisfactory (Con.)*  *wished for an extension of the telephone hours (Con.)* |  |
|  | Für die Mitarbeiterinnen, die am Telefon beraten, finden regelmäßig Supervisionen und Fortbildungen (intern und extern) statt. | | Supervisionen und Fortbildungen (Org.) |  |  |
|  | *Supervision and further training (internal and external) take place on a regular basis for the staff members who provide counselling on the telephone.*  *(translated by authors)* | | *Supervision and further training (Org.)* |  |  |
| ALZ i-Connect [36] | Clients appreciate the Association staff reaching out to them especially during this often confusing and stressful time following a diagnosis. | |  |  | Clients appreciate (…) reaching out to them (Con.) |
|  | 90% found that the presented information was clear and helpful. | |  | Information was clear and helpful (Con.) |  |
|  | 81% of responders reported feeling comfortable during the skype session. | |  |  | feeling comfortable (Con.) |
| Care Consultation Plus [38] | When asked to rate the helpfulness of action steps, more than 80 % of all callers reported action steps to be helpful. Furthermore, 70 % of all callers had reported putting action steps in place at one week following the baseline consultation. | |  | reported action steps to be helpful (Con.) |  |
| Helpline Alz Ass East Massa [40] | Family Support Group information was reported as "helpful" to "extremely helpful" in 80 percent of the cases. Long term careplanning information received a 100 percent positive rating response, perhaps indicating the more proactive callers are those who perceive the helplines as most beneficial. | |  | Long term careplanning information received a 100 percent positive rating response (Con.) | was reported as „helpful“ to “extremely helpful” (Con.) |
|  | A summary variable was created to reflect the overall helpfulness of information provided by the helpline. This variable was used to determine the  strength of the relationship between the extent to which the caller provides caregiving and their perception of the overall helpfulness of the information  provided. Using bivariate statistics, a strong inverse relationship (r = -.51, p < .05) was found between the number of hours spent caregiving and the caller's perception of helpfulness. Thus, those who spent fewer hours caregiving experienced the helpline more positively. | |  | helpfulness of the information provided (Con.) |  |
|  | The results of the study indicate a high level of overall satisfaction with the helpline. | |  |  | high level of overall satisfaction (Con.) |
|  | While overall satisfaction with the helpline was high, callers who received follow-up information through the mail tended to report greater satisfaction. The majority (87 percent) noted they would call the helpline again as concerns arose in the future. | |  | received follow-up information (…) to report greater satisfaction (Con.) | call the helpline again (Con.) |
| Sabat^a^ [43] | This information was key in helping Mrs U to understand her husband’s condition more clearly, interact with him more effectively than she had previously and, thereby, gain a measure of control over what was happening in their lives. Subsequently, she took a series of actions that resulted in her experiencing great growth and satisfaction. | |  |  | Experiencing great growth and satisfaction (Con.) |
|  | Though no attempt is made to generalize from this case study, it is nevertheless clear that: (1) education and psychosocial support via email resulted in a positive evolution in Mrs U’s experience as a caregiver (...) | |  |  | resulted in a positive evolution (…) as a caregiver (Con.) |
| Salfi^a^, ^b^ [44–46] | However, they enjoyed the satisfaction of empowering caregivers to cope with difficult situations. | |  |  | Satisfaction of empowering caregivers to cope (Prov.) |
|  | The major theme of caregiver experiences with telephone support related to a sense of companionship and connection. Caregivers talked about having a “lifeline” and someone at the other end of the telephone: | |  |  | Sense of a companionship and connection (Con.) |
|  | They described the comfort of knowing that someone is out there who will listen to them, acknowledge their efforts, and provide encouragement and reassurance: | |  |  | Comfort (…) who will listen (…), acknowledge their efforts (Con.) |
| FITT-C [47–53] | At the end of the intervention, caregivers in both conditions were asked about satisfaction with the intervention. Overall satisfaction rates (1 5 not satisfied to 4 5 very much satisfied) did not significantly differ between the groups (FITT-C M 5 3.83, SD 5 .51 vs. TS M 5 3.78, SD 5 .49 P 5.57). | |  |  | Overall satisfaction rates (…) did not significantly differ (Con.) |
| FITT-D [54] | To address treatment satisfaction, caregivers in the FITT-D group completed a 12-item treatment satisfaction questionnaire at the end of treatment. Caregivers reported a high level of satisfaction across all domains. | |  |  | high level of satisfaction (Con.) |
|  | Most caregivers gave the intervention the highest possible rating for the following categories: satisfied with quality of service (94%), kind of service wanted (82%), meeting needs (77%), recommend to friend (88%), amount of help (82%), deal with problems effectively (82%), therapist skills (100%), convenience (94%), written materials (88%), and clear and understandable (94%). For overall satisfaction, 94 percent of caregivers reported that they were very much satisfied. Most caregivers (88%) reported that they would be very likely to seek similar treatment again. | |  | therapist skills (100%), convenience (94%), written materials (88%), and clear and understandable (94%) (Con.) | satisfied with quality of service (94%), kind of service wanted (82%), meeting needs (77%), recommend to friend (88%), amount of help (82%), deal with problems effectively (82%) (Con.)  overall satisfaction (Con.) |
| NVAMP [55] | When caregivers’ concerns were feelings of stress and strain, their opportunity to share these feelings with the nurse were seen as reasons the telephone calls were helpful (Table 1). In other words, the reasons the conversations were viewed as helpful were dependent on the initial stress and strain experienced by caregivers. | |  |  | feelings of stress and strain (…) were helpful (Con.) |
|  | Major perceived reasons for helpfulness were that participants were assisted in sharing thoughts and feelings, expressing feelings of being overwhelmed, discussing physical and psychosocial problems, forgetting the situation, seeking reassurance, and asking for information; several participants perceived the telephone calls to be lacking in helpfulness. | |  |  | perceived reasons for helpfulness (Con.)  Perceived the telephone calls to be lacking in helpfulness (Con.) |
|  | Caregivers in both groups welcomed the opportunity to vent their negative feelings of being overwhelmed. | |  |  | welcomed (…) vent their negative feelings of being overwhelmed (Con.) |
|  | However, this study indicates caregivers more often perceived a compassionate ear as being helpful. | |  | Compassionate ear as being helpful (Con.) |  |
|  | Focus group data highlighted user satisfaction with the online support and reliability of the environment. | |  |  | Satisfaction with the online support (Con.) |
| ICSS [56–61] | Caregivers felt the online therapists were professional, caring, and provided satisfactory advice. | |  | therapists were professional, caring, and provided satisfactory advice (Con.) |  |
|  | felt that the therapists would respect and understand the importance of their cultural values. | |  |  | would respect and understand the importance of their cultural values (Con.) |
|  | Frequent users who were interviewed felt the service was satisfactory and helpful because it provided practical suggestions, allowed them to express their feelings, and increased their competence to give care. | |  |  | felt the service was satisfactory and helpful (Con.) |
|  | Some preferred English because they had been in Canada for a long time. Most preferred to type in English because typing Chinese on an English keyboard was difficult. They liked to hear the stories of other caregivers. Participants preferred to be notified when there was new information, and they welcomed the idea of receiving notices in their regular email account, such as their Hotmail and Yahoo accounts. | |  | preferred to be notified when there was new information (Con.)  welcomed the idea of receiving notices (Con.) | Preferred to type in English (Con.) |
|  | Participants who had used the e-mail support felt they could express themselves freely and relieve their stress in e-mails. (...) Caregivers said they would not use the e-mail support when they felt they could handle the care. (...) Even if they did not send any e-mail, they felt good that they had something to fall back on when there was a problem. Some felt that writing in English did not allow them to express themselves fully. Others were unsure what to ask or how to ask. | |  |  | could express themselves freely (Con.)  had something to fall back on (Con.)  writing in English did not allow them to express themselves fully (Con.) |
| InformCare [62–64] | All participants in the three countries generally had a positive and satisfying experience with the platform, | |  |  | Positive and satisfying experience (Con.) |
| Link2Care [65] | Client satisfaction surveys reveal that two-thirds of caregivers found Link2Care helpful in their efforts to become more knowledgeable about caregiving issues. | |  |  | found Link2Care helpful in their efforts (Con.) |
|  | Eighty-six percent said they would recommend Link2Care to other caregivers. | |  |  | would recommend (Con.) |
|  | The top five features valued by Link2Care users are: updated news and research, information articles and fact sheets, online discussion groups, “Ask the Expert,” and local educational event listings. | |  | Updated news and research, information articles and fact sheets, online discussion groups, “Ask the Expert,” and local educational event listings (Con.) |  |
| Online Coaching Program [66] | in 2008,94% (81% in 2006) of survey respondents agree that they feel better prepared to cope with present and future caregiving issues. | |  |  | feel better prepared to cope with present and future caregiving issues (Con.) |
| De Cola^a^ [67] | The videoconferencing service provided was well appreciated by the elderly and their caregivers/relatives, with regard to the tele-counseling. In particular, they felt less lonely and more cared for while the caregivers were better able to cope with the relatives’ illness. | |  |  | was well appreciated (Con.)  were better able to cope (Con.) |
|  | Indeed, the mean SUS scoring (68.9) indicated a high rate of efficacy and satisfaction of the users, taking into account that scores greater than 68 are considered above average, and only in 10% in the studies such a score exceeds 80.32 | |  |  | high rate of efficacy and satisfactions (Con.) |
| Laver^a^ [68,69] | Overall, participants reported moderate-to-high levels of satisfaction with the program although participants allocated to receive home visits appear to provide somewhat more favorable responses. | |  |  | moderate-to-high-level of satisfaction with the program (Con.) |
| Dementelcoach [74–79] | Overall, the informal caregivers valued the telephone coaching with a mean score of 8.31 (SD = 1.08, range 1–10), indicating that informal caregivers were indeed highly satisfied with the telecoaching they received in the intervention period. | |  |  | highly satisfied (Con.) |
|  | Overall, the informal caregivers were satisfied to very satisfied with the different aspects of the intervention, in particular the friendliness of the coaches, the interest coaches showed in the problems of caregivers, and the way the coaches communicated with the caregivers. In addition, caregivers said they valued having someone who listened to them: they felt they could be really frank with their coaches and tell them things anonymously that they would not easily share with others.  s. a. Table 3 and 4 | |  | friendliness of the coaches, the interest coaches showed in the problems of caregivers, and the way the coaches communicated with the caregivers (Con.) |  |
|  | The large majority proved satisfied or very satisfied with the coaching (40%, 40%) and the STAR e-Learning course (64%, 29%). One person was unsatisfied and one moderately satisfied about the coaching and one person was moderately satisfied with the STAR e-Learning. (...) The groups gave a mean grade of 7.8 (SD=1.48) for Dementelcoach, 7.8 (SD=0.98) for STAR e-Learning, and 8.4 (SD=0.73) for regular MCSP (on a scale from 1 to 10). | |  |  | satisfied or very satisfied with the coaching (Con.) |
| Nomura^a^ [80] | In conclusion, participation in the PAR was a deeply satisfying experience for all team members. | |  |  | deeply satisfying experience for all team members (Prov.) |
|  | However, there were families who did not appreciate the benefits of the PAR. Those who were too busy with work/ child-rearing or lived far away from the PWD failed to detect small changes in the PWD. Regardless of the FCPs’ degree of participation, FCPs asked for counselling when the PWD’s psycho-behavioural symptoms worsened. | |  |  | did not appreciate the benefits of par (Con.) |

*Abbreviations*: ADS = Alzheimer's Disease Society; Con. = Consumer; FITT-C = Family Intervention: Telephone Tracking – Caregiver; FITT-D = Family Intervention: Telephone Tracking – Dementia; Helpline Alz Ass East Massa = Helpline of the Alzheimer's Association of Eastern Massachusetts; ICSS = Internet-based Caregiver Support Service; NVAMP = Nurse Video With Assisted Modeling Program; Org. = Organization; Prov. = Provider

*Notes*: ^a^When no name is reported, the name of the first author was assigned to the intervention.

^b^Assignment of the quote(s) to experimental and comparator intervention not possible, we assume, the information is applicable for both interventions.

## Table S4: Analysis matrix for ‘adoption’

| **Original definition of ‘adoption’ according to Proctor et al. [17]:**  “*Adoption* is defined as the intention, initial decision, or action to try or employ an innovation or evidence-based practice.” | | **Adaptation of the definition:**  *Adoption* is defined as the intention, initial decision, or action to try or employ a technology-based counselling intervention. | | | | |
| --- | --- | --- | --- | --- | --- | --- |
| **Intervention^a^** | **Quote** | | **Dimensions of ‘adoption’ (level of analysis)** | | | |
|  |  |  | **Organizational motive** | **Mode of decision** | **Uptake of interventions motivated** | |
|  |  |  |  |  | **internally** | **externally** |
| Admiral Nurse Dementia Helpline [29–32] | Dementia UK is a charity committed to helping families face dementia. Its network of Admiral Nurses provides specialist one-to-one support and expert advice to help families “cope with the fear, uncertainty and difficult everyday reality of dementia” (www.dementiauk.org/how-we-help/). | | committed to helping families face dementia (Org.) |  |  |  |
| Alz i-connect [36] | The Cleveland Area Chapter was looking for an alternative way to replicate the personal connection, increase efficiency, and reduce costs and staff time away from the office. The solution was the creation of ALZ i-Connect, a unique approach to connect individuals who have just received a diagnosis to important information about memory loss and the Association’s free services before they even leave their physician’s office. | |  |  | was looking for an alternative way to replicate the personal connection, increase efficiency, and reduce costs and staff time away from the office (Org.) |  |
|  | The Alzheimer’s Association Cleveland Area Chapter has implemented a novel approach for individuals to connect to helpful information about Alzheimer’s disease and related dementias while at the physician’s office. | | for individuals to connect to helpful information (Org.) | Alzheimer’s Association Cleveland Area Chapter has implemented a novel approach (Org.) |  |  |
| CANDID [37] | However, once the diagnosis has been established, and the illness is progressing, the need for specialist neurological investigation decreases, while the need for support and advice increases; yet by this stage patients and carers find it increasingly difficult to travel to an outpatient clinic in London.  To resolve some of these problems, and to provide a source of support that is available throughout the illness, the CANDID (Counselling and Diagnosis in Dementia) service was launched in February 1995. | | provide a source of support that is available throughout the illness (Org.) | To resolve some of these problems (Org.) was launched (Org.) | need for specialist neurological investigation decreases, while the need for support and advice increases; yet by this stage patients and carers find it increasingly difficult to travel (Sett.) |  |
| Helpline Alz Ass East Massa [40] | This study examined the helpline of the Alzheimer's Association of Eastern Massachusetts. Its stated mission is to provide access to comprehensive services as well as provide support to callers who are caring for someone with dementia. | | to provide access to comprehensive services as well as provide support (Org.) |  |  |  |
| ODCC [42] | The association for people with dementia and their families in Okayama, Japan, is a local public interest incorporated association, which established a telephone support system for dementia, named ODCC, entrusted by both of Okayama city and Okayama prefecture in June 2011. | |  | local public interest incorporated association, established a telephone support system for dementia (…) entrusted by both of Okayama city and Okayama prefecture (Org.; Admin.) |  |  |
|  | The staffs belonging to ODCC were dedicated to listen to the problems of clients and caregivers, to provide them with useful information, and to carefully advise them regarding necessary medical consultations | | dedicated to listen to the problems (…), to provide them with useful information, and to carefully advise them (Org.) |  |  |  |
| FITT-C [47–53] | Many caregivers encounter barriers that make in-person interventions difficult, including lack of transportation, being homebound, living in a rural setting, time pressures of caregiving, or stigma associated with seeking help. In response to these issues, our group developed the Family Intervention: Telephone Tracking—Caregiver (FITT-C) | |  | In response to these issues, our group developed (Org.) |  | caregivers encounter barriers that make in-person interventions difficult (Sett.) |
|  | Compared with standard care, we found that an earlier version of FITT showed reductions in perceived burden and less-severe reactions to memory and behavior problems [17]. | |  |  | an earlier version of FITT showed reductions in perceived burden and less-severe reactions to memory and behavior problems (Org.) |  |
| FITT-D [54] | The FITT model was initially identified as a potentially effective intervention for dementia caregivers because of preliminary data showing efficacy for stroke caregivers (Miller et al., 1998). | |  |  | identified as a potentially effective intervention for dementia caregivers because of preliminary data showing efficacy for stroke caregivers (Org.) |  |
| Link2Care [65] | Family Caregiver Alliance, a research and advocacy group in San Francisco, California, conceived of the idea as they become aware of a combination of factors: increasing use of the Internet (now in 60 percent of U.S. house-holds), availability of more sophisticated information (with use of such vehicles as Medline online searches, for example), a downward trend in the age of first-time callers seeking help from Family Caregiver Alliance (currently, 60 percent are age 64 and under) and more caregivers in the workforce—that lead to examining options for using the Internet to assist families with caregiving. | |  |  | conceived of the idea as they become aware of a combination of factors (Org.)  that lead to examining options (Org.) | increasing use of the Internet (…), availability of more sophisticated information (…), a downward trend in the age of first-time callers seeking help from Family Caregiver Alliance (…) and more caregivers in the workforce (Sett.) |
|  | As they face caregiving challenges within their own circle of family and friends, more and more individuals, especially baby boomers and younger age groups, are turning to the Internet for information about health, treatment of diseases, care management, current research findings, and mutual support. (…) It should come as no surprise, then, that Link2Care, an innovative Internet-based program to provide information and support to family caregivers, has been a definite success in the years since its inception in 1999. | |  |  |  | more and more individuals, especially baby boomers and younger age groups, are turning to the Internet for information (Sett.) |
|  | It was these questions and others that lead us to investigate the world of consumer health informatics and e-health technologies to look for the promising practices and determine how they could be adapted to the area of caregiving and management of chronic care. Fortunately, we found a small but growing body of experience on Internet-based technologies on which to build. | |  |  |  | look for the promising practices and determine how they could be adapted to the area of caregiving and management of chronic care (Org.)  small but growing body of experience (…) on which to build (Org.) |
| De Cola^a^ [67] | In this scenario, innovative local and national initiatives, designed to meet the increased demand for health care services, are taking place18 in an attempt to reduce the number of older people admitted in residential care homes and hospitals.19 Thus, for the first time ever in Southern Italy, the IRCCS Centro Neurolesi ‘Bonino-Pulejo’ of Messina together with the Sicilian government have included a telehealth system within a family-centred care programme | | meet the increased demand for health care services (…) to reduce the number of older people admitted in residential care homes and hospitals (Org.) | for the first time ever (…) the IRCCS Centro Neurolesi ‘Bonino-Pulejo’ of Messina together with the Sicilian government have included a telehealth system within a family-centred care programme (Org., Sett., Admin.) |  |  |
| Laver^a^ [68,69] | Programs showing beneficial effects have been conducted over five to ten sessions and delivered in the home.10 One such intervention (“Care of Persons with dementia in their Environments” [COPE]) was found to be effective in a large randomized controlled trial (n = 237) conducted in the United States and led by Gitlin and colleagues.11 | |  |  |  | One such intervention (…) was found to be effective (Org.) |
| Dementelcoach [74–79] | First the centres were recruited among members of the National Working Group of Meeting Centres: all centres in the Netherlands received an email in which they were invited to participate in the project. Centres who were willing to do so, and had been operational for at least 1.5 years, were eligible for the study. | |  | Centres who were willing to do so (Org.) |  |  |
| Nomura^a^ [80] | Permission to implement the programme was obtained from the executive administrator of the rural town involved | |  | Permission to implement the programme was obtained (Admin.) |  |  |
|  | In the early 1990s, the local public health nurses (PHNs) conducted a survey on the living conditions of people with dementia and their families and found that some demented elderly residents were compelled to stay indoors due to their disease. | | living conditions of people with dementia and their families (Org.) |  |  |  |
|  | In 1998 the PHNs initiated various types of activities, such as dementia prevention education and mental health counselling for the elderly. | |  | PHNs initiated various types of activities (Org.) |  |  |

*Abbreviations*: Admin. = Administration; CANDID = Counselling and Diagnosis in Dementia; FITT-C = Family Intervention: Telephone Tracking – Caregiver; FITT-D = Family Intervention: Telephone Tracking – Dementia; Helpline Alz Ass East Massa = Helpline of the Alzheimer's Association of Eastern Massachusetts; ODCC = Okayama Dementia Call Center; Org. = Organization; Prov. = Provider; Sett. = Setting

*Note*: ^a^When no name is reported, the name of the first author was assigned to the intervention.

## Table S5: Analysis matrix for ‘appropriateness’

| **Original definition of ‘appropriateness’ according to Proctor et al. [17]:**  “*Appropriateness* is the perceived fit, relevance, or compatibility of the innovation or evidence based practice for a given practice setting, provider, or consumer; and/or perceived fit of the innovation to address a particular issue or problem.” | | | **Adaptation of the definition:**  *Appropriateness* is the perceived fit, relevance, or compatibility of the technology-based counselling intervention for the given practice setting, provider, and consumer; and/or perceived fit of the intervention to address a particular issue or problem. | | | |
| --- | --- | --- | --- | --- | --- | --- |
| **Intervention^a^** | **Quote** | **Dimensions of ‘appropriateness’ (level of analysis)** | | | | |
|  |  | **Overall compatibility with stakeholder needs** | | **Tailoring to individuals** | **Skills and instruments for enhancing fit** | **Concepts for fit** |
| Admiral Nurse Dementia Helpline [29–32] | The helpline has responded to some of the aforementioned challenges by offering a range of ways to access support, such as by telephone, email, social media and directly via the Dementia UK website. | offering a range of ways to access support, such as by telephone, email, social media and directly via the Dementia UK website (Org.) | |  |  |  |
|  | These interventions can include delivering psychosocial interventions, such as person-centred approaches to interaction and communication and environmental modifications (Johnston and Narayanasamy, 2016), as well as providing advice and information bespoke to the caller’s needs. |  | | bespoke to the caller’s needs (Prov.) |  | person-centred approaches to interaction and communication (Prov.) |
|  | Admiral Nurses work holistically to consider the needs of the whole family affected by dementia. |  | |  |  | work holistically to consider the needs of the whole family (Prov.) |
|  | The helpline nurse employs specialist communication skills to engage the caller to make a rapid assessment and appraisal of their need to then offer the most appropriate intervention. This can be a complex process, particularly if the caller has contacted the helpline in a distressed or anxious state (Pollock et al, 2010). |  | | the most appropriate intervention (Prov.) | specialist communication skills (Prov.)  assessment and appraisal of their need (Prov.) |  |
|  | Much of the Admiral Nurse role is in empowering family carers to seek various supports they need but often this may require varying degrees of active involvement on a case-by-case basis. |  | | on a case-by-case basis (Prov.) |  |  |
|  | The nurses on the helpline are required to use a bespoke range of approaches, all drawn from evidence-based practice and delivered using a person-centred approach. |  | |  |  | evidence-based practice (Prov.)  person-centred approach (Prov.) |
|  | It offers dedicated support from an Admiral Nurse via telephone or email and operates seven days a week, between 9 am to 9 pm Monday to Friday and 9 am to 5 pm on Saturday and Sunday. | via telephone or email and operates seven days a week, between 9 am to 9 pm Monday to Friday and 9 am to 5 pm on Saturday and Sunday (Org.) | |  |  |  |
|  | The nurse used counselling skills to explore and validate John's feelings and concerns, enabling John to express his and his mother's distress. |  | | enabling John to express his and his mother's distress (Prov.) | counselling skills to explore and validate (…) (Prov.) |  |
|  | The nurse sourced the latest evidence and information (...), while being aware of the challenges callers can experience in absorbing and retaining large amounts of information. | being aware of the challenges callers can experience in absorbing and retaining large amounts of information (Prov.) | |  |  |  |
|  | Guided discovery was used to explore John's thoughts and to encourage him to consider a strategy to reduce his distress and assist him to support his father. (...) The nurse used Socratic questioning to prompt John to reflect on his views and beliefs (...) The nurse and John explored the reasons why John may not been consulted on decisions made about his father's care (...) |  | |  | Guided discovery (Prov.)  Socratic questioning (Prov.) |  |
|  | The nurse discussed with John a suggested script that he could use at an upcoming meeting with the multidisciplinary team caring for his father planned for later that week. (...) In John's case, the suggested script included statements that he felt comfortable making when talking to the hospital team (...). The script also included a discussion of treatment options for Joseph (...). After the call, the nurse summarised its details in an email to John. |  | | the suggested script included statements that he felt comfortable making (Prov., Con.)  nurse summarised its details in an email (Prov.) | suggested script (Prov.) |  |
|  | During John's description of the situation, the nurse conducted a biopsychosocial assessment using the Admiral Nurse assessment framework. Following the assessment, the nurse provided a response specific to John's situation, informed by his needs and including the issues important to him, thereby putting him in control. |  | | nurse provided a response specific to John's situation (Prov.) | biopsychosocial assessment (Prov.) |  |
|  | The role of the helpline is also to ensure that the caller can use the information provided to them effectively. | ensure that the caller can use the information (…) effectively (Prov.) | |  |  |  |
|  | John's case showed how important it is for nurses running the helpline to continuously refine their assessment skills, which are crucial when attempting to prioritise issues in a short space of time. Generally, nurses have only one interaction with callers, so the ongoing development of their skills in processing large amounts of information, determining priorities and providing meaningful support is crucial. |  | |  | assessment skills (Prov.)  skills in processing large amounts of information, determining priorities and providing meaningful support (Prov.) |  |
|  | As the telephone helpline is operated by skilled Admiral Nurses they use their specialist knowledge and experience to effect the best approach for each caller and their individual circumstances. |  | | best approach for each caller and their individual circumstances (Prov.) | specialist knowledge and experience (Prov.) |  |
|  | The helpline operates seven days and five evenings a week and is manned by trained Admiral Nurses, who also provide an email service. | operates seven days and five evenings a week  also provide an email service (Org.) | |  |  |  |
|  | Wilkinson (2016) noted that ANs on the Direct helpline were relatively unusual in that they do not strive for neutrality but that one of their ‘hallmarks’ was the high degree of understanding and empathy displayed as part of the process of providing emotional support for family carers. In my conversation with Michael it was important that he felt emotionally supported so I used responses, such as, ‘I do understand how you feel’ and ‘I know what you’re going through’. This is an important element of enabling the caller to tell you their problems and that you understand. |  | |  | enabling the caller to tell you their problems (Prov.) | understanding and empathy displayed as part of the process of providing emotional support (Prov.) |
|  | more than three-quarters of calls to the Admiral Nurse Dementia Helpline involved giving ‘bespoke’ advice to the caller |  | | ‘bespoke’ advice to the caller (Prov.) |  |  |
|  | Tailoring a script to a carer, based on his/her individual situation and particular needs, is known as ‘recipient design’ (Wilkinson, 2011). |  | | Tailoring a script to a carer (Prov.) |  | ‘recipient design’ (Prov.) |
|  | Most of the calls (N=243; 80%) included discussion of the difficulties the caller was experiencing on a day-by-day basis with the PWD for whom they were caring (see ‘banner headlines’ in Part (i) above for typical problems). In response to these day-by-day difficulties, one distinctive practice frequently employed by call-takers was to suggest specific practical and/or psychological strategies to help the caller manage the situation. |  | | discussion of the difficulties the caller was experiencing (Prov., Con.) |  | practical and/or psychological strategies (Prov.) |
|  | Offering a ‘script proposal’ (Emmison, Butler and Danby, 2011) involves giving a caller an example of what they might say in a given situation (in effect, a ‘script’ they might follow). The script is presented in the first person – i.e. the call-taker ‘voices’ the words of the caller in order to ‘model’ what he or she might possibly say. |  | | call-taker ‘voices’ the words of the caller in order to ‘model’ what he or she might possibly say (Prov.) | ‘script proposal’ (Prov.) |  |
| ADS helpline [33] | The main issues discussed included the help that is available, the assessment process, the first signs of Alzheimer's disease and treatable causes. Other callers wanted information about genetics or about what would happen to them and their family. Furthermore, some were concerned that their GP was not taking their concerns seriously or that they had not had a proper assessment. |  | | main issues discussed (…) Other callers wanted information (…) some were concerned (Con.) |  |  |
| Alzheimer helpline [34,35] | Die Bandbreite der Fragen und Probleme, mit denen Angehörige durch die Demenzerkrankung in der Familie befasst sind, ist nach wie vor sehr groß. In fast jedem Beratungsgespräch werden mehrere Themen angesprochen. |  | | In fast jedem Beratungsgespräch werden mehrere Themen angesprochen. (Prov.) |  |  |
|  | *The range of questions and problems relatives are dealing with due to dementia in the family is still very wide. Several topics are addressed in almost every counselling session.*  *(translated by authors)* |  | | *Several topics are addressed in almost every counselling session. (Prov.)* |  |  |
|  | Sprechzeiten montags bis donnerstags von 9 bis 18 Uhr und freitags von 9 bis 15 Uhr | Sprechzeiten (Org.) | |  |  |  |
|  | *Office hours Monday to Thursday from 9 a.m. to 6 p.m. and Friday from 9 a.m. to 3 p.m.*  *(translated by authors)* | *Office hours (Org.)* | |  |  |  |
|  | Das Alzheimer-Telefon ist ein anonymes Angebot, das heißt niemand muss seinen Namen oder seine Adresse angeben, es sei denn die Zusendung von Imformationsmaterialien wird gewünscht. | niemand muss seinen Namen oder seine Adresse angeben (Org.) | |  |  |  |
|  | *The Alzheimer's telephone is an anonymous service, which means that no one has to give their name or address unless they wish to receive information material.*  *(translated by authors)* | *no one has to give their name or address (Org.)* | |  |  |  |
|  | Obwohl nach wie vor der überwiegende Anteil der Ratsuchenden das Telefon als Kontaktmedium nutzt (81%), hat der Anteil der Anfragenden per E-Mail im Jahr 2006 erneut zugenommen und beträgt 17% (Vorjahr 13%). (...) Die Chat-Beratung, die seit Mai 2003 ergänzend angeboten wird, wird nur in Einzelfällen wahrgenommen. | Telefon als Kontaktmedium (…) Anfragenden per E-Mail (...) Chat-Beratung (Org.) | |  |  |  |
|  | *Although the majority of those seeking counselling still use the telephone as a means of contact (81%), the percentage of those requesting counselling by e-mail increased again in 2006 and amounts to 17% (previous year 13%). (...) Chat counselling, which has been offered as a supplement since May 2003, is only used in individual cases.*  *(translated by authors)* | *telephone as a means of contact (…) those requesting counselling by e-mail (...) Chat counselling (Org.)* | |  |  |  |
|  | Da jede dieser schwierigen Verhaltensweisen individuelle Ursachen und Folgen haben kann, ist eine spezifische Beratung notwendig. Deshalb wird es auch in Zukunft ein wichtiges Ziel der Berater/innen am Alzheimer-Telefon sein, den anfragenden Personen weiterführende Hilfen und Ansprechpartner in ihrer Region zu nennen |  | | spezifische Beratung (Prov.) |  |  |
|  | *Since each of these difficult behaviours can have individual causes and consequences, specific counselling is necessary. For this reason, it will continue to be an important goal of the counsellors at the Alzheimer's telephone to inform the inquiring persons of further help and contact persons in their region.*  *(translated by authors)* |  | | *specific counselling (Prov.)* |  |  |
|  | Dass der Anteil von Kindern und Schwiegerkindern bei den Email-Anfragen größer ist, ist nicht verwunderlich aufgrund der größeren Computeraffinität der jüngeren Generation. | größeren Computeraffinität der jüngeren Generation (Sett.) | |  |  |  |
|  | *The fact that the share of children and children-in-law in email enquiries is larger is not surprising due to the greater computer affinity of the younger generation.*  *(translated by authors)* | *greater computer affinity of the younger generation (Sett.)* | |  |  |  |
| Alz i-connect [36] | During the Skype call, staff will coordinate a follow-up time to speak (ideally within 3-7 days) with the patient or family members to talk in further detail about the initial information shared, answer new questions, and engage them in clinical services as needed. |  | | follow-up time to speak (…) with the patient or family members to talk in further detail about the initial information shared, answer new questions, and engage them in clinical services as needed (Prov.) |  |  |
|  | ALZ i-Connect allows users to connect face-to-face with Alzheimer’s Association staff via Skype technology in a private setting at the doctor’s office, right after receiving their diagnosis. This initial conversation between patient/caregiver and an Alzheimer’s disease professional gives the diagnosed individual and his or her family an opportunity to ask many questions they have about memory and thinking disorders. This intervention allows the Alzheimer’s Association to share facts about the disease and resources available. | connect face-to-face with Alzheimer’s Association staff via Skype technology (Org.) | | gives the diagnosed individual and his or her family an opportunity to ask many questions (Con., Prov.) |  |  |
|  | 81% reported this audiovisual call was beneficial. 72% thought that the ALZ i-connect call was an efficient way of communicating and gathering information from the Association, while only 18% would have preferred a different means of communication with the association. | audiovisual call (Org.)  an efficient way of communicating and gathering information  would have preferred a different means of communication (Con.) | |  |  |  |
| CANDID [37] | A central aim of CANDID is that it should be more than a simple source of information, and that it should have the ability to influence and alter the care and management of younger patients with dementia. Moreover, it should have a holistic approach, liaising with professionals and family members, providing advice on the practical, social and legal issues of these diseases as well as the medical aspects. Finally, the service aims to be truly ‘CANDID' with patients and carers, encouraging open discussion about diagnosis and the sharing of information. | aims to be truly ‘CANDID' with patients and carers, encouraging open discussion (Org.) | |  |  | holistic approach (Prov.) |
|  | However, once the diagnosis has been established, and the illness is progressing, the need for specialist neurological investigation decreases, while the need for support and advice increases; yet by this stage patients and carers find it increasingly diffcult to travel to an outpatient clinic in London. | by this stage patients and carers find it increasingly diffcult to travel to an outpatient clinic (Con.) | |  |  |  |
|  | CANDID differs from other services by aiming to provide medical advice and intervention at a distance, which can be specific to the individual and be targeted at either the patient, carer, GP or other healthcare professional. | provide medical advice and intervention at a distance (Org.) | | specific to the individual and be targeted at either the patient, carer, GP or other healthcare professional (Org.) |  |  |
|  | CANDID has no age barrier, but has been tailored to the needs of patients where the disease has started before the age of 65 years and promoted for this group of people and their carers. | has been tailored to the needs of patients where the disease has started before the age of 65 years (Org.) | |  |  |  |
|  | CANDID is such a service, which serves a small population of patients and their carers who are distributed across the UK. | serves a small population of patients and their carers (Org.) | |  |  |  |
|  | The service is primarily a telephone helpline, though enquiries can also be made in person, by post and by electronic mail (e-mail). In addition to providing advice and support by telephone, the nurse/counsellors see carers of patients on the wards and a small number of carers who attend in person, having had an initial telephone consultation | primarily a telephone helpline, though enquiries can also be made in person, by post and by electronic mail (e-mail) (Org.) | |  |  |  |
|  | CANDID represents a new concept in providing care for a group of patients with rare diseases. | providing care for a group of patients with rare diseases (Org.) | |  |  |  |
| Care Consultation Plus [38] | The follow up calls, however, need to be tailored to individual needs as well as based on notes from previous discussion(s). |  | | tailored to individual needs as well as based on notes from previous discussion(s) (Org., Prov.) |  |  |
|  | also received an additional follow up call from the same care consultant to reinforce specific action steps and identify barriers to following through on the action steps. |  | | from the same care consultant to reinforce specific action steps (Prov.) |  |  |
| Care Consultation / Care Consultation Plus^b^ [38] | It is well suited to individuals who may be homebound with limited time and energy to seek formal supportive services. Additionally, the availability of a 24/7 Helpline means caregivers can access support at the time of need. | well suited to individuals who may be homebound (Org., Sett.)  availability of a 24/7 helpline (Org.)  caregivers can access support at the time of need (Con.) | |  |  |  |
|  | Care consultation begins with an assessment of the caller’s situation and provides in-depth education about the disease and problem solving that result in the development of an action plan. (...) The caller’s needs, identified through the assessment, determine the type of intervention that is appropriate. |  | | assessment of the caller’s situation  caller’s needs (…) determine the type of intervention that is appropriate (Prov.) |  |  |
|  | The Alzheimer’s Association Helpline (referred to as the Helpline in this paper) is an easily accessible and free resource available 24 h a day, 365 days a year, where callers can speak to master’s degree-level care consultants who offer confidential emotional support, valuable and actionable information and referrals to additional resources in the local community. | easily accessible and free resource available 24 h a day, 365 days a year (Org.) | |  |  |  |
| Coyne^a^ experimental intervention [39] | During each "extended contact" telephone call, subjects were engaged in further discussion of the caregiving issues they were dealing with; they were offered additional information, advice, and literature; |  | | further discussion of the caregiving issues they were dealing with (Con., Prov.) | literature (Prov.) |  |
| Coyne^a^, ^b^ [39] | caller was encouraged to talk about any dementia-related issues he or she was concerned about; all questions were answered and requests for advice responded to; and services were made |  | | was encouraged to talk about any dementia-related issues he or she was concerned about (Con., Prov.) |  |  |
|  | a toll- free, New Jersey-statewide helpline providing information, referral, education, and counseling for dementia- related issues | a toll- free, (..) statewide helpline (Org.) | |  |  |  |
|  | staffed during regular working hours by one of the authors with assistance from other full-time COPSA staff | regular working hours (Org.) | |  |  |  |
|  | Helpline staff can provide a large measure of support and encouragement through counseling techniques which emphasize the relief of caregiver stress as a primary goal. At the same time, staff must avoid adding to a caregiver's burden by making him or her feel pressured to follow up on resource information provided. |  | |  | counseling techniques (Prov.)  staff must avoid adding to a caregiver's burden by making him or her feel pressured (Prov.) |  |
| Helpline Alz Ass East Massa [40] | Table 4 provides a breakdown of the information provided in response to the individual caller's stated and/or implied need. |  | | response to the individual caller's stated and/or implied need (Prov.) |  |  |
|  | The majority of callers (67 percent) who reported receiving mailed information, read the materials completely. | mailed information (Org.) | |  |  |  |
|  | In response to questions regarding any new concerns that may have come up since the caller's contact with the helpline, 37 percent (37) affirmed that they did have new concerns. About 27 percent (10) of these respondents had called the helpline again. | they did have new concerns (…) had called the helpline again (Con.) | |  |  |  |
|  | The findings of this study suggest that those individuals most likely to access the helpline are those with an inadequate support network. Of the respondents in this survey, 46 percent stated that the Alzheimer helpline was the first place they turned for support. | individuals most likely to access the helpline are those with an inadequate support network (Sett.)  helpline was the first place they turned for support (Sett.) | |  |  |  |
|  | However, helpline callers who did receive mailings from the Alzheimer's Association, reported some difficulty in distinguishing between the many pamphlets and brochures. They had trouble recalling which were helpful, and which were not helpful pieces of information. | callers who did receive mailings (…) reported some difficulty in distinguishing between the many pamphlets and brochures (Con.) | |  | pamphlets and brochures (Prov.) |  |
|  | That is, telephone services provided from 9 a.m. to 5 p.m. | telephone services provided from 9 a.m. to 5 p.m. (Org.) | |  |  |  |
|  | Of the 100 callers who were included in this study, 37 percent (37) had one or more suggestions for improvements to the helpline. Suggestions included 19 percent (7) requests for programs to create greater public awareness. Another 16 percent (6) felt there should be more information provided for specific geographic regions. About 8 percent (3) recommended the establishment of an Alzheimer support telephone network, particularly to reach isolated caregivers who may be unable to attend support group meetings. | suggestions for improvements to the helpline (Con.) | |  |  |  |
| Natale^a^ [41] | a neuropsychologist and a trained geriatrician were available for questions, focusing on education‐behavior problem‐solving strategies and practical advices |  | | available for questions (Prov.) | practical advices (Prov.) | education‐behavior problem‐solving strategies (Prov.) |
| ODCC [42] | In this telephone support system, experienced experts that included family or public caregivers of dementia patients accepted a variety of consultations by phone calls from clients who are mainly the family members of dementia patients, while assuring anonymity. The staffs belonging to ODCC were dedicated to listen to the problems of clients and caregivers, to provide them with useful information, and to carefully advise them regarding necessary medical consultations. | consultations by phone calls (Org.) | | dedicated to listen to the problems of clients and caregivers (Prov.) |  |  |
|  | The fact that the proportion of giving advice decreased as the proportion of listening increased, indicates the difficulties in solving problems among family. Tailored advice for each client by ODCC may be essential for clear decision making and to provide appropriate care, and can lead to a reduction in a caregiver’s burden of psychological distress and finally reduce patients’ symptoms.6–8 |  | | Tailored advice for each client (Prov.) |  |  |
|  | the present study showed that children or children-in-law called more than spouses, suggesting that old partners might hesitate to consult ODCC phone service than their children. | children or children-in-law called more than spouses (Sett.) | |  |  |  |
| Sabat^a^ [43] | In other words, I did not choose the areas to address in this process. Rather, it was the caregiver’s needs and frustrations as she expressed them in her ongoing correspondence that dictated the areas on which I focused attention. Thus, she essentially ‘led’ the process and I responded to her in kind. |  | | the caregiver’s needs and frustrations (…) dictated the areas on which I focused attention (Prov.) |  |  |
| Salfi^a^ nonanonym [44–46] | The provider affiliated with the organization that offered nonanonymous telephone support noted that knowing the caregiver and his or her situation was definitely a strength of their telephone support service, as it assisted her to better meet the needs of the caregivers. | knowing the caregiver and his or her situation was definitely a strength (Prov.) | | to better meet the needs of the caregivers (Prov.) |  |  |
|  | One caregiver whose father attended the Adult Day Program (associated with the multiservice, telephone support agency) and who had met with the provider face-to-face described her preference for speaking with someone who was aware of her situation and personally knew her father: | preference for speaking with someone who was aware of her situation (Con.) | |  |  |  |
|  | Some providers in the study had contact with caregivers as part of a multicomponent support programme. These providers understood the caregiver’s situation when offering support. | providers understood the caregiver’s situation (Prov.) | |  |  |  |
| Salfi^a^ anonym [44–46] | Those providing support without knowing the caregiver and having little understanding of the specific caregiving context experienced frustration and difficulties. | providing support without knowing the caregiver and having little understanding of the specific caregiving context experienced frustration and difficulties (Prov.) | |  |  |  |
| Salfi^a^, ^b^ [44–46] | Fifth, careful consideration should be given to the advantages and disadvantages of an anonymous telephone support intervention. Knowing about the caregiver/care recipient situation prior to providing telephone support was viewed as helpful by both caregivers and providers, and having the ability to follow-up with caregivers was important to all of the providers, especially after difficult support sessions. This is not possible with an anonymous, caregiver-initiated service, which may be an option preferred by some caregivers. | advantages and disadvantages of an anonymous telephone support (Org.)  Knowing about the caregiver/care recipient situation prior to providing telephone support was viewed as helpful (Con., Prov.)  ability to follow-up (Prov.)  caregiver-initiated service, which may be an option preferred by some caregivers (Org., Con.) | |  |  |  |
|  | Although it was revealed that current caregiver interventions such as educational sessions, support groups, and respite are all helpful, telephone support was described as an intervention that has the ability to meet the various needs of caregivers, in the comfort of their own home. | telephone support (…) has the ability to meet the various needs of caregivers (Org.) | |  |  |  |
|  | Most importantly, telephone support can be advantageous at any stage in the caregiving cycle, from initial selection of community supports, throughout the process of long term care placement, to the final stages of grief and bereavement. | telephone support can be advantageous at any stage in the caregiving cycle (Org.) | |  |  |  |
|  | All of the caregivers interviewed identified the need for telephone support to be offered beyond the 8-hour workday, especially during the final stages of the illness. Four of the eight caregivers described the need for a 24-hour service. | need for telephone support to be offered beyond the 8-hour workday (…) need for a 24-hour service (Con.) | |  |  |  |
|  | Caregivers appreciated the convenience of telephone support because they could access it immediately from home, without worrying about booking appointments or finding substitute care for their family members. Telephone support was comfortable in that it could be used as a single resource for different types of problems, and it continued after the person with dementia moved to institutional care. | access it immediately from home, without worrying about booking appointments or finding substitute care (Con.)  resource for different types of problems (Con.) | |  |  |  |
|  | Limitations of telephone support included restricted hours and lack of service for non-English speaking clients. | restricted hours and lack of service for non-English speaking clients (Con.) | |  |  |  |
|  | Both agencies offered information and emotional support tailored to individualized needs via the telephone intervention. |  | | tailored to individualized needs (Prov.) |  |  |
|  | Caregivers described how telephone support assisted them with making linkages within the system: | assisted (.) with making linkages within the system (Con.) | |  |  |  |
|  | Caregivers and providers recognized that the convenience of telephone support was an important dimension of this intervention. Caregivers could remain in their homes without having to leave the care recipient while they accessed this service. | could remain in their homes without having to leave the care recipient (Con.) | |  |  |  |
|  | The findings from this study also revealed two caregiver needs that were not being met by current telephone support services: (a) the need for increased hours of availability and (b) the need for a multilingual service. | need for increased hours of availability (…) need for a multilingual service (Con.) | |  |  |  |
|  | The loss of context associated with a telephone interaction was also described as a cause of helplessness. Providers described this loss of context as not being able to see how the caregiver was reacting to or receiving their advice or input and not being able to physically respond to the caregiver’s reactions (especially when they were upset or angry). | loss of context as not being able to see how the caregiver was reacting to or receiving their advice or input and not being able to physically respond to the caregiver’s reactions (Prov.) | |  |  |  |
|  | Both caregivers and telephone support providers described the intervention of telephone support as a source of information and educational advice, tailored to each individual caregiving situation. Caregivers described their receipt of advice, strategies, or tools to effectively manage and cope with problematic situations. | receipt of advice, strategies, or tools to effectively manage and cope with problematic situations (Con.) | | tailored to each individual caregiving situation (Con., Prov.) |  |  |
|  | Both groups agreed that the experience of telephone support was more rewarding when caregivers and providers had met face to face and when the provider knew the person with dementia. | experience of telephone support was more rewarding when caregivers and providers had met face to face and when the provider knew the person with dementia (Con.) | |  |  |  |
| FITT-C [47–53] | The second stage, Follow-up, involves telephone follow-up contacts in which any new problems are identified, positive and negative changes in caregivers or patients are discussed, psychoeducational information is reviewed and applied for a particular situation, and assistance is provided to help the caregiver solve problems. |  | | new problems are identified, positive and negative changes in caregivers or patients are discussed (Con., Prov.)  for a particular situation (Prov.)  assistance is provided to help the caregiver solve problems (Prov.) | psychoeducational information (Prov.) |  |
|  | Therapists can choose the most appropriate intervention from a menu of choices, including supportive approaches (i.e., empathy, giving permission, normalizing, provision of information, validation, or venting) or more active strategies (i.e., bibliotherapy, interpretation, positive reframing, problem solving, reference to resource packet, referral, or setting task directives). |  | | the most appropriate intervention (Prov.) |  | supportive approaches (i.e., empathy, giving permission, normalizing, provision of information, validation, or venting) or more active strategies (i.e., bibliotherapy, interpretation, positive reframing, problem solving, reference to resource packet, referral, or setting task directives) (Prov.) |
|  | FITT-C was designed to be flexible enough to assist caregivers whose care recipient has a predominance of neuropsychiatric, cognitive, or behavioral symptoms. |  | | flexible (Prov.) |  |  |
|  | After the final call, the therapist prepares a letter that briefly highlights the progress during the intervention and encourages the caregiver to continue to develop and utilize adaptive coping strategies. |  | |  | letter that briefly highlights the progress (Prov.) adaptive coping strategies (Prov.) |  |
|  | Each caregiver was assigned one therapist, who made all telephone contacts with that caregiver. |  | | Each caregiver was assigned one therapist (Org.) |  |  |
|  | One factor that may have enhanced retention in the study was that the telephone was used for intervention implementation and assessments. We took a number of steps to improve the intervention delivery by telephone (e.g., bios and pictures of therapists) and assessments (e.g., providing response formats). | telephone was used for intervention implementation and assessments (Org.) | |  | improve the intervention delivery by telephone (e.g., bios and pictures of therapists) and assessments (e.g., providing response formats) (Prov.) |  |
|  | The sample is mainly Caucasian, which limits generalizability to other racial and ethnic groups. We made attempts to increase diversity in the sample through community outreach, but were limited by several factors, especially the inclusion criterion that required a formal dementia diagnosis by a specialist. There is evidence that racial and ethnic minorities have inadequate access to specialty care and are influenced by cultural factors about the effects of normal aging and mistrust of the medical establishment | sample is mainly Caucasian (…) racial and ethnic minorities have inadequate access to specialty care (Sett.) | |  |  |  |
|  | an entirely telephone-delivered caregiver intervention, to enhance accessibility (...). | entirely telephone-delivered caregiver intervention, to enhance accessibility (Org.) | |  |  |  |
|  | Despite our best recruitment efforts, the sample was mainly Caucasian, so we are unable to generalize findings to ethnically and racially diverse groups. There is evidence that different ethnic groups may respond differently to caregiver interventions [9]. | different ethnic groups may respond differently to caregiver interventions (Sett.) | |  |  |  |
|  | Initial calls were 60 minutes long, and follow-up calls were 15 to 30 minutes long, depending on the severity of caregiver problems. |  | | depending on the severity of caregiver problems (Prov.) |  |  |
|  | Although the FITT-C could be easily adapted into an internet-based intervention or implemented by video-conferencing technology, the simplicity and widespread availability of the telephone has clear advantages. | simplicity and widespread availability of the telephone (Org.) | |  |  |  |
| FITT-D [54] | Furthermore, although the intervention is manualized and structured, it maintains considerable flexibility to allow for individualized application of interventions as deemed necessary and appropriate. |  | | individualized application of interventions as deemed necessary and appropriate (Prov.) |  |  |
|  | The second stage, Follow-up, involved weekly and bi-weekly contacts in which new problems were identified, positive and negative changes in caregivers or care recipients were discussed, and psychoeducational information was reviewed and applied for particular situations. |  | | new problems were identified, positive and negative changes in caregivers or care recipients were discussed (Con., Prov.) | psychoeducational information (Prov.) |  |
|  | This is the first study to show that an entirely telephone-based intervention can reduce dementia caregiver burden. | entirely telephone-based (Org.) | |  |  |  |
|  | The length of the intervention may also be important. Most caregiver interventions are six months or less. Although the telephone contacts were generally short (less than 30 minutes), FITT-D was delivered over one year. This provided an opportunity for the therapist to help caregivers apply problem-solving skills in a variety of situations and in the face of cognitive and behavioral decline in the care recipients. | over one year (…) opportunity for the therapist to help caregivers apply problem-solving skills in a variety of situations and in the face of cognitive and behavioral decline in the care recipients (Org., Prov.) | |  |  |  |
|  | In the current study, we chose to continue treating caregivers after placements. We believed that the FITT-D was adaptable enough to be effective with these caregivers. |  | | adaptable (Prov.) |  |  |
| NVAMP [55] | During the follow-up telephone calls, the problem-solving process for the intervention consisted of identifying the concern or problem, sources of difficulty, possible sources of support, and alternatives for addressing problems. Nurses then conversed with caregivers about the various options and the pros and cons of the options available to them. Caregivers were assisted in reframing their concerns where possibilities were appropriate. |  | | Nurses then conversed with caregivers about the various options and the pros and cons of the options available to them (Prov.) |  | problem-solving process  reframing their concerns (Prov.) |
|  | Assisting caregivers through the problem-solving process with some of the conditions they were facing was part of the NVAMP intervention program. Thus, it was not surprising that all of the caregivers in the experimental group expressed the help they received as being one of the reasons they found the telephone calls to be useful (Table 2). | found the telephone calls to be useful (Con.) | |  |  | problem-solving process (Prov.) |
|  | Finally, some caregivers indicated the telephone support was not useful or complained about the standardized measurements (Table 2). Only one caregiver from the experimental group indicated the information was not applicable; however, in subsequent conversations, this caregiver indicated she tried some of the strategies and found them to be useful. | support was not useful (Con.)  tried some of the strategies and found them to be useful (Con.) | |  |  |  |
| ICSS [56–61] | Caregivers were given an email account to email a therapist at a time that was convenient to them, using a language of their choice. | to email a therapist at a time that was convenient to them, using a language of their choice (Con.) | |  |  |  |
|  | After the caregivers had activated the account, they could start using the email support service. Online therapists sent a welcome message to them and invited them to describe their caregiving concerns. The email communication was customized to meet the needs of each caregiver. |  | | invited them to describe their caregiving concerns  customized to meet the needs of each caregiver (Prov.) |  |  |
|  | Participants who had used the e-mail support felt they could express themselves freely and relieve their stress in e-mails. (...) Caregivers said they would not use the e-mail support when they felt they could handle the care. (...) Even if they did not send any e-mail, they felt good that they had something to fall back on when there was a problem. Some felt that writing in English did not allow them to express themselves fully. Others were unsure what to ask or how to ask | writing in English did not allow them to express themselves fully (Con.)  unsure what to ask or how to ask (Con.) | |  |  |  |
|  | This technology-based program was designed exclusively for Chinese Canadians with diverse ethno-cultural-linguistic backgrounds. | technology-based program  exclusively for Chinese Canadians with diverse ethno-cultural-linguistic backgrounds (Org.) | |  |  |  |
|  | These findings suggest that Chinese caregivers may need support services tailored to their ethnic-cultural belief systems. The ICSS system was built to address these issues and to provide easy accessibility through the use of technology. | tailored to their ethnic-cultural belief systems (Org., Sett.)  easy accessibility through the use of technology (Org.) | |  |  |  |
|  | The therapists initiated the first e-mail contact, introduced themselves, and asked each participant to share any concerns they had about caring for a family member with dementia. The e-mail exchange occurred asynchronously in a language of choice (English, Simplified Chinese, or Traditional Chinese). | e-mail exchange occurred asynchronously in a language of choice (Con., Prov.) | | asked each participant to share any concerns they had about caring for a family member with dementia (Prov.) |  |  |
|  | this study used asynchronous e-mails, enabling the caregivers to access service at a time convenient to them | asynchronous e-mails, enabling the caregivers to access service at a time convenient to them (Org.) | |  |  |  |
|  | this study assigned a professional to each caregiver for continuity of service. The ongoing communications facilitated rapport building and minimized the need for the caregivers to repeat telling their situations. |  | | assigned a professional to each caregiver for continuity of service (Org.) |  | rapport building (Prov.) |
|  | Depending on caregiver needs, each therapist provided information about the disease and its management, such as strategies for ensuring home safety and strategies for managing the care recipient’s difficult behaviour. |  | | Depending on caregiver needs, each therapist provided information (Prov.) |  |  |
|  | Delivering support services via ICT mediation seems to involve a complex interaction between the caregiver’s capacity to use the Internet and the caregiver’s needs for service. The findings showed non-users were older and were more competent in giving care while frequent users were younger and rated themselves as less competent. | non-users were older and were more competent in giving care while frequent users were younger and rated themselves as less competent (Con.) | |  |  |  |
|  | In the interviews, frequent users felt that the email mode of communication was useful because it enabled them to express their concerns freely and receive immediate support from a health professional. They felt the information site was comprehensive and helpful, with all the information they needed in one place. Non-users or occasional users explained the challenges they experienced. Although Chinese caregivers needed informational and emotional support, addressing these needs using online bilingual support was not sufficient to ensure service use – users’ English or computer literacy had an impact on the perception of service usefulness. Their usage pattern was affected by their information seeking behaviour and learning styles. Inputting Chinese language was a challenge. Some forgot to access the service. | email mode of communication was useful (Con.)  occasional users explained the challenges they experienced (Con.)  users’ English or computer literacy had an impact on the perception of service usefulness (Con.) | |  |  |  |
|  |  |  |  |  |  |  |
|  | E-mail contact was the only form of communication between the therapists and caregivers throughout the program. | E-mail contact was the only form of communication (Org., Prov., Con.) | |  |  |  |
|  | Also, traditional beliefs shaped caregivers’ needs, and ethnocultural-linguistic contexts affected system usability and were associated with usage behavior. | ethnocultural-linguistic contexts affected system usability and were associated with usage behavior (Sett.) | |  |  |  |
|  | In this study, client-centered concepts have been applied when designing the service (i.e. the use of e-mail as the medium) and providing the intervention (i.e. the content and process of intervention). |  | |  |  | client-centered concepts (Org.) |
|  | E-mail contact allows caregivers to choose a time that is convenient to them without the need to make appointments. | choose a time that is convenient to them without the need to make appointments (Org.) | |  |  |  |
|  | It was decided that the intervention would be personalized, flexible and be provided by occupational therapists who were familiar with the cultural background of Chinese Canadians. | therapists who were familiar with the cultural background (Prov.) | | personalized, flexible (Prov.) |  |  |
|  | Caregivers would decide when to communicate, how much to tell, and which language to use (Chinese or English). Information, education, and emotional support were provided based on the expressed needs of the caregivers. | Caregivers would decide when to communicate, how much to tell, and which language to use (Con.) | | provided based on the expressed needs of the caregivers (Prov.) |  |  |
|  | The caregivers were encouraged to share their questions, express their emotion, and discuss their viewpoints on the caregiving concerns important to them. They were actively involved in identifying solutions, acting upon them, and evaluating the outcomes of the strategies. |  | | encouraged to share their questions, express their emotion, and discuss their viewpoints on the caregiving concerns important to them (Prov.) |  |  |
|  | The e-mail communication medium supported a flexible, individualized service the caregivers wanted. The content analysis showed that the caregivers decided how much to write, what to write, and when to write. They are ultimately responsible for their choices and decisions.The analysis of the communication process between the caregivers and therapists showed that although the process did not involve face-to-face interaction, it was similar in many respects to what occurs in home- or hospital-based interventions with family caregivers. The online therapists employed conventional person-centred communication techniques such as validation, empathic understanding, and encouragement (Baptiste, 2010) to develop rapport and build a trusting relationship with each caregiver. |  | | flexible, individualized service the caregivers wanted (Prov.)  caregivers decided how much to write, what to write, and when to write (Con.) | to develop rapport and build a trusting relationship with each caregiver (Prov.) | conventional person-centred communication techniques such as validation, empathic understanding, and encouragement (Prov.) |
|  | Analysis of the therapists’ e-mail responses showed that they employed strategies frequently used in face-to-face intervention programs for dementia caregivers. These strategies include validation, empathic understanding, and clarification of communication. |  | |  |  | validation, empathic understanding, and clarification of communication (Prov.) |
|  | Caregivers who were actively engaged in the service valued the individualized, flexible service that enabled their performance of caregiving occupation. |  | | individualized, flexible service (Con.) |  |  |
|  | Many caregivers have to balance competing responsibilities while fulfilling their care-giving role. Some worked full time, while having other roles and responsibilities, such as being a mother, homemaker, daughter, and so on. Only caregivers who could juggle these various roles could spare time to access and use the services. For instance, one caregiver was unable to find time to access the service when her job demands increased substantially. For several months because she had to work overtime and take work home, she did not have the time to use the service until the job situation returned to normal. Another caregiver described that it was not easy to manage her time when she had full-time work, was a homemaker and a mother of two teenage children, and took care of her parents who both have health needs (her mother has dementia). She said she had a supportive husband who took primary responsibility for taking care of the children. She hired a helper for the household chores and to help take care of her mother. She coordinated the care for her mother among the helper, her father, and her brother. Because this particular caregiver was able to juggle her competing roles, she managed to find time in the late evenings to use the support intervention. | Only caregivers who could juggle these various roles could spare time to access and use the services. (Con.) | |  |  |  |
|  | Caregivers felt that the perceived efforts to use the technology seemed to be greater when they were under stress, which made them more reluctant to use the support intervention. | perceived efforts to use the technology seemed to be greater when they were under stress (Con.) | |  |  |  |
|  | (b) Usability: We followed the recommendations published by the National Institute on Aging ( 2001 ) to make the Web site senior friendly (Chiu & Henderson, 2005 ) and applied general usability principles to achieve an ease-of-use layout, including format and comprehension of posted content (short paragraphs, grades six to eight reading level) (Brinck, Gergle, & Wood, 2002). | make the Web site senior friendly  ease-of-use layout, including format and comprehension of posted content (Org.) | |  |  |  |
|  | During the meetings, the therapists reported that they needed to spend more time to “read between the lines” so as to understand the subtle emotions embedded in the e-mail messages. |  | |  | to “read between the lines” so as to understand the subtle emotions embedded in the e-mail messages (Prov.) |  |
| InformCare [62–64] | The analysis suggested the intervention was useful and appropriate, also stimulating a better self-efficacy and reappraisal of the caregivers’ role. | intervention was useful and appropriate (Con.) | |  |  | self-efficacy and reappraisal of the caregivers’ role (Prov.) |
|  | Focus group data highlighted user satisfaction with the online support and reliability of the environment. | reliability of the environment (Con.) | |  |  |  |
|  | Both group and individual support provided by professional counselors was considered optimal and brought clear benefits. | support provided by professional counselors was considered optimal (Con.) | |  |  |  |
|  | On the other hand, qualitative findings from the focus groups pointed out the usefulness and appropriateness of support received by caregivers from information and communication with moderators and peers. | usefulness and appropriateness of support (Con.) | |  |  |  |
|  | Despite the majority of participants who judged the usability of the platform as sufficient or good, some of them did mention technical or usability issues as a reason for not having used some of the available interactive services more. In particular, Swedish users reported problems with using the mobile version and specific features of some services (eg, uploading pictures on the social network, and using the chat and videochat features), whereas in Italy some caregivers found it difficult to find and reach some internal pages or services. In Sweden, an alternative videocommunication system was used with the moderator in order to overcome technical issues arising with the videochat feature. Support guaranteed by the moderator was in any case highly appreciated by all users across the three countries. | usability of the platform as sufficient or good (Con.)  technical or usability issues as a reason for not having used (Con.) | |  |  |  |
|  | Caregivers used private messages and e-mails when they needed specific support from the professional | private messages and e-mails when they needed specific support (Con.) | |  |  |  |
|  | The great majority of participants also reported that the Web program was useful for addressing both current and possible future caregiving needs. | Web program was useful (Con.) | |  |  |  |
|  | the emerging opinion was that these kinds of services solve time and logistical issues compared with conventional services (eg, face-to-face meetings). Furthermore, because they were always available, interactive services were perceived as particularly flexible. Nevertheless, two participants from Italy and Sweden added that it would be preferable to have them integrated with physical meetings, to sustain personal contacts and gain support that is not available or possible to receive online. Swedish participants pointed out that it was good to have both open services (eg, social network and forum) and private ones (eg, private messages), to combine support from different sources. Generally speaking, being in contact with other caregivers appeared to be simpler and quicker. Across countries, participants agreed that having a professional moderator was particularly important to stimulate interaction and for prompt, tailored support. | solve time and logistical issues compared with conventional services (Con.)  always available (…) particularly flexible (Con.)  preferable to have them integrated with physical meetings, to sustain personal contacts and gain support that is not available or possible to receive online (Con.) | |  |  |  |
|  | Finally, participants reported that there was a set of“ missing pieces” in the context of the Web program. First, digital skills training for caregivers was perceived as a priority by Italian participants, who had less knowledge of and experience with Web services. They felt that such training would help increase their confidence with using the interactive services both within the platform and in general Web navigation. Second, Italian and Swedish participants pointed out a need to broaden the repertoire of services by including other types of professional support and learning, such as physicians and nurses. Dedicated virtual desks or online appointments with them would be helpful for getting more tailored health information, advice, and counseling on clinical aspects of the care recipient. | set of“missing pieces” in the context of the Web program (Con.) | |  |  |  |
| Link2Care [65] | More than 60 percent of caregivers surveyed reported Link2Care to be useful in coping with caregiving and reducing feelings of isolation. Some 80 percent found the Internet availability to be very helpful. | to be useful in coping with caregiving and reducing feelings of isolation (Con.)  Internet availability (Org.) | |  |  |  |
| Online Coaching Program [66] | Online Coaching was developed to provide individualized support and information to caregivers. Often it is difficult for caregivers to find the time and respite care needed to attend traditional support groups or meetings. Many are familiar with the concept of personal coaching as it relates to career development, personal growth, physical fitness, and healthy living. |  | | individualized support and information to caregivers (Prov.) |  | concept of personal coaching (Prov.) |
|  | In most circumstances, the coach replied to correspondence within 24 hours. When the coach e-mailed or replied to a correspondence, the participants received an automated notification via their work or home e-mail informing the participant that a message was waiting on their member page. Participants, many of whom were still working full time, had the convenience of contacting their coach when their schedule allowed, and the coach was able to provide information on disease process and caregiving in a timely manner through the resource library. | coach replied to correspondence within 24 hours  received an automated notification (Prov.)  convenience of contacting their coach when their schedule allowed (Con.) | |  | resource library (Prov.) |  |
|  | Clients' use of the site has varied dramatically (ranging front 1 login to 1327 logins per client). Some program participants were very utilitarian in their use of the Web site and communication with their coach. They would use the site primarily as a tool to access information on caregiving and disease process. Other clients were more interested in using the site for emotional support, journaling, and accessing library resources and the health center. These clients wrote more frequently and in much more depth about their emotions surrounding their caregiving situation. For both groups of participants, the coach was able to meet their needs and respond accordingly. | use of the site has varied dramatically (Con.)  use the site primarily as a tool to access information (…) in using the site for emotional support, journaling, and accessing library resources and the health center (Con.) | |  |  |  |
|  | This program used a secure Web site to provide individualized support and information to those caring for individuals with dementia |  | | individualized support and information (Prov.) |  |  |
|  | In 2008, 100% (100% In 2006) of survey respondents agree that their coach offered support and assistance when they most needed it. In 2008. 100% (100% in 2006) of survey respondents agree that the information they received was, timely and on target with their needs. | coach offered support and assistance when they most needed (Prov.)  timely and on target with their needs (Con.) | |  |  |  |
| De Cola^a^ [67] | The main feature of our system, compared to others, is that it offers the possibility to monitor patients in a more personalized way, by giving psychological and medical counseling either to the subjects or to their caregivers. |  | | monitor patients in a more personalized way, (…) either to the subjects or to their caregivers (Prov.) |  |  |
|  | The videoconferencing service provided was well appreciated by the elderly and their caregivers/relatives, with regard to the tele-counseling. | well appreciated by the elderly and their caregivers/relatives, with regard to the tele-counseling (Org., Con.) | |  |  |  |
| Laver^a^ [68,69] | When interpreting the findings, it is critical to reiterate that trial participants who were allocated to receive telehealth intervention commenced the intervention program with two visits from an occupational therapist in the home environment. These two visits may have been crucial for establishing rapport and develop a platform to continue the therapeutic relationship. Furthermore, these first two visits to the home offered the therapist the ability to properly assess and understand the home environment and observe the relationship and interactions between the person with dementia and their family members. Delivering the program in its entirety via telehealth may influence uptake and the therapeutic relationship. | two visits from an occupational therapist in the home environment (…) may have been crucial for establishing rapport (…) to properly assess and understand the home environment and observe the relationship and interactions between the person with dementia and their family members (Org., Prov.) | |  |  | therapeutic relationship (Prov.) |
|  | Strategies to address key care challenges are tailored to the capabilities and interests of the person with dementia, their care partner and the environment. |  | | are tailored to the capabilities and interests of the person with dementia, their care partner (Prov.) |  |  |
| RCTM [70–73] | The RCTM similarly offers individual and family counseling, ad hoc support, and knowledge and skills transfer to help families adapt to RLTC [62]. The RCTM is a semi-structured intervention and tailored to address the individual needs and concerns of a primary family caregiver. | individual and family counseling, ad hoc support (Org.) | | tailored to address the individual needs and concerns of a primary family caregiver (Prov.) |  |  |
|  | Together, TCs and family caregivers identify individual placement stressors and enhance caregivers’ strategies for coping with them. |  | | individual placement stressors and enhance caregivers’ strategies (Prov.) |  | strategies for coping (Prov.) |
|  | The RCTM incorporates psychosocial and psychoeducational approaches with the objective of: a) focusing on the identification of potential stressors associated with RLTC placement for caregivers; and b) assisting caregivers to develop more effective individual coping strategies and enhanced caregiving self-efficacy following the institutionalization transition. |  | |  |  | psychosocial and psychoeducational approaches (Prov.)  coping strategies  self-efficacy (Prov.) |
|  | Ad-hoc sessions may be added at any time | Ad-hoc sessions may be added at any time (Org.) | |  |  |  |
|  | The RCTM is not limited by geographic distance, allowing for flexible delivery. The individualized, tailored content of the RCTM that addresses key content areas and specific areas of need (regardless of time since admission to RLTC) is another feature that likely enhances its implementation potential as the intervention can meet the heterogeneous needs of families following RLTC admission of a relative. | not limited by geographic distance, allowing for flexible delivery (Org.) | | individualized, tailored content (Prov.) |  |  |
|  | Tele-health: Telephone or secure web conference calls are placed to location of primary caregiver’s preference (often their home; cell phone use is common, so location is frequently varied) | Telephone (…) web conference calls (…) cell phone use is common (Org., Prov., Con.) | | are placed to location of primary caregiver’s preference (Prov.) |  |  |
|  | Primary caregivers participated in counseling and additional supporting family members were included at the primary caregiver’s request. |  | | additional supporting family members were included at the primary caregiver’s request (Con., Prov.) |  |  |
|  | We identify specific opportunities for tailored couple and family psychology interventions, including communication strategies, decision-making approaches, focusing on positives, psychoeducation, self-forgiveness exercises, stress management and self-care activities, and validation. The current work informs how counseling interventions can provide practical support by highlighting specific clinical mechanisms that help to alleviate common facets of caregiver guilt following a transition into RLTC. Critically, we distinguish variation between spouses and adult children to design treatment plans that best support clients who are caring for a person living with dementia in RLTC. |  | | distinguish variation between spouses and adult children to design treatment plans that best support clients (Prov.) | treatment plans (Prov.) | couple and family psychology interventions, including communication strategies, decision-making approaches, focusing on positives, psychoeducation, self-forgiveness exercises, stress management and self-care activities, and validation (Prov.) |
|  | The semi-structured intervention was tailored to the needs and preferences of individual caregivers. The TC offered session topics including guilt, grief, conflict resolution, stress management, and psychoeducation. The sessions centered on building therapeutic rapport with the caregiver and providing a safe environment to explore stressors, caregiving roles, and family dynamics relating to quality of life for the caregiver and care recipient. The TC helped the caregiver explore their guilt or shame around placement, perceptions of their care recipient’s reaction to the transition, and life in RLTC. | centered on building therapeutic rapport (Prov.)  providing a safe environment (Prov.) | | tailored to the needs and preferences of individual caregivers (Prov.)  helped the caregiver explore (Prov.) |  | guilt, grief, conflict resolution, stress management, and psychoeducation (Prov.) |
|  | individualized sessions by phone or secure Web-based video conferencing (…) Other family members were also included in the counseling sessions based on the needs expressed by the primary caregiver. | by phone or secure Web-based video conferencing (Org.) | | individualized sessions (Prov.)  family members were also included in the counseling sessions based on the needs expressed by the primary caregiver (Con., Prov.) |  |  |
|  | The RCTM is a semi-structured individualized intervention, allowing caregivers to choose which session topics they would like to discuss and when. |  | | allowing caregivers to choose which session topics they would like to discuss and when (Prov.) |  |  |
|  | In addition to the six sessions, the TC was available to the caregiver throughout their time in the study for ad hoc contact or counseling sessions as requested by the caregiver. | TC was available to the caregiver throughout their time in the study for ad hoc contact or counseling sessions as requested by the caregiver (Org.) | |  |  |  |
|  | In these instances, the TC provided validation of the caregiver’s concerns and helped them brainstorm solutions. For caregivers with specific upcoming one-on-one conversations with staff or care conferences, the TC coached caregivers by providing suggestions and structure to address concerns in the conversation. |  | |  | TC provided validation of the caregiver’s concerns and helped them brainstorm solutions (Prov.) | validation (Prov.) |
|  | The RCTM intervention was tailored to the individual needs of each caregiver regardless of setting |  | | was tailored to the individual needs of each caregiver (Prov.) |  |  |
|  | Although quantitative findings were limited in scope and explanation, qualitative analysis of counseling sessions demonstrate that the RCTM provided real-time opportunities for caregivers to raise concerns about communities and staff and receive coaching, suggestions, and plans to improve communication and their experience with the community. | provided real-time opportunities for caregivers to raise concerns (Org.) | |  |  |  |
| Dementelcoach [74–79] | covering issues caregivers wanted to discuss |  | | issues caregivers wanted to discuss (Con.) |  |  |
|  | Informal caregivers in our study wanted more personal contact with their coach (meeting face-to-face at least once) and reported wishing to have the possibility of contacting the coach in a crisis situation. | caregivers (…) wanted more personal contact with their coach (Con.)  wishing to have the possibility of contacting the coach in a crisis situation (Con.) | |  |  |  |
|  | Telephone support could offer an alternative solution for those who are not making optimum use of the care services: the intervention enables caregivers to receive support in their own personal environment, making it easily accessible to many caregivers, including not only those who are unable to travel but also those who have no other services available nearby. Telephone coaching has the added value of providing personalized support that is tailored to the specific problems experienced by caregivers. | receive support in their own personal environment (Con.)  making it easily accessible (Org.) | | tailored to the specific problems experienced by caregivers (Prov.) |  |  |
|  | Professional caregivers were trained as telephone coaches to provide informal caregivers the appropriate support and, if relevant, to refer them to other care and support services that met their needs (van Mierlo et al., 2012). |  | |  | trained (…) to provide informal caregivers the appropriate support (Prov.) |  |
| Nomura^a^ [80] | Staff routinely asked about any changes and problems faced and allowed caregivers to talk about care burden issues. On these occasions, the staff educated the caregivers about dementia for them to understand the PWD’s behaviours and what to expect. For example, many FCPs complained that the PWD tended to lose precious items. After this behaviour was explained, a typical response was ‘‘I became upset many times as she lost very expensive things, now I understand that because it is precious to her, she tried to hide in a place where no one would look for it.’’ The counselling programme aimed to coach problem-focused coping. |  | | allowed caregivers to talk about care burden issues (Prov.) |  | problemfocused coping (Prov.) |
|  | Counselling was offered face-to-face on request from the beginning of the PAR. Then, phone counselling was started in the second year as a part of the monthly phone interviews, and counselling was offered when a problem was identified by the FCP. | offered when a problem was identified by the FCP (Con., Prov.) | |  |  |  |
|  | Through the increased use of phone counselling, problems related to PsWD were identified at an early stage, as shown in the conversation log sheets, and the need for face-to-face counsellings gradually decreased (Table 2). | Through the increased use of phone counselling, problems (…) were identified at an early stage  need for face-to-face counsellings gradually decreased (Con.) | |  | conversation log sheets (Prov.) |  |
|  | Third, the content of the monthly phone interviews and counsellings with caregivers was recorded in a communication log sheet. The caregivers reported the PWD’s conditions as well as their own feelings and behaviour towards the PWD. If the FCP reported any problem, a counselling was offered and recorded in the log sheet. |  | | If the FCP reported any problem, a counselling was offered (Con., Prov.) | communication log sheet (Prov.) |  |
|  | In our study, staff counsellors played the role of coach to assist FCPs to identify the major problem and to discuss the strategies together. ‘‘Coaches are trained to listen, to observe and to customize their approach to individual client needs. They seek to elicit solutions and strategies from the client; they believe the client is naturally creative and resourceful’’ (International Coach Federation, 2007). |  | |  | the role of coach to assist FCPs  trained to listen, to observe and to customize their approach to individual client needs (Prov.) |  |

*Abbreviations*: ADS = Alzheimer's Disease Society; CANDID = Counselling and Diagnosis in Dementia; Con. = Consumer; FITT-C = Family Intervention: Telephone Tracking – Caregiver; FITT-D = Family Intervention: Telephone Tracking – Dementia; Helpline Alz Ass East Massa = Helpline of the Alzheimer's Association of Eastern Massachusetts; ICSS = Internet-based Caregiver Support Service; NVAMP = Nurse Video With Assisted Modeling Program; ODCC = Okayama Dementia Call Center; Org. = Organization; Prov. = Provider; RCTM = Residential Care Transition Module; Sett. = Setting

*Notes*: ^a^When no name is reported, the name of the first author was assigned to the intervention.

^b^Assignment of the quote(s) to experimental and comparator intervention not possible, we assume, the information is applicable for both interventions.

## Table S6: Analysis matrix for ‘feasibility’

| **Original definition of ‘feasibility’ according to Proctor et al. [17]:**  “*Feasibility* is defined as the extent to which a new treatment, or an innovation, can be successfully used or carried out within a given agency or setting (Karsh 2004).” | | **Adaptation of the definition:**  *Feasibility* is defined as the extent to which the technology-based counselling intervention can be successfully used or carried out within a given agency or setting. | | | |
| --- | --- | --- | --- | --- | --- |
| **Intervention^a^** | **Quote** | | **Dimensions of ‘feasibility’ (level of analysis)** | | |
|  |  |  | **Practicability** | **Factors impeding feasibility** | **Factors promoting feasibility** |
| Admiral Nurse Dementia Helpline [29–32] | Case management and the coordination of care are central to the role of an Admiral Nurse in the community (Harrison Dening, Aldridge, Pepper, & Hodgkison, 2017) but is often a skill called upon on the telephone helpline. I researched online and identified that there was an emergency home care service nearby. I was able to telephone them up and clarify that they would accept a self-referral from a family carer and could respond the same day if required. | | I researched online and identified (Prov.) I was able to telephone them up and clarify (Prov.) |  |  |
|  | Equally, the lack of visual cues can mean that essential information required for a holistic clinical assessment are not possible (Geddes et al, 2020). This means that the nurse has to draw on their specialist training to manage the expectations of the caller and mitigate the impact of their emotional or psychological distress. This invariably requires enhanced assessment skills of counselling and psychological approaches via the telephone. | | essential information required for a holistic clinical assessment are not possible (Prov.) | lack of visual cues (Prov.) | specialist training to manage the expectations of the caller and mitigate the impact of their emotional or psychological distress (Prov.) |
| Alz i-connect [36] | The Cleveland Clinic as a way to give assessment center staff the opportunity to connect families right away with the free support services available at the Association. The Alzheimer’s Association chapter provides a TV with camera to the site location, which is then connected to the center’s Internet service. Assessment Center staff receive brief training from the Association on the use of the equipment and how to easily connect their patients to the Alz i-connect service via Skype. | | a way to give (…) the opportunity (Sett.)  provides a TV with camera to the site location (Org.)  to easily connect (Org., Sett.) |  | receive brief training from the Association (Sett., Org.) |
|  | Since Skype is not a Health Insurance Portability and Accountability Act-compliant information technology platform, this exchange is not meant to extract personal information about the patient or their medical history. If the patient and/or their family would like a more indepth conversation related to their personal situation, a follow-up phone call will be scheduled at this time. | |  | is not a Health Insurance Portability and Accountability Act-compliant information technology platform (Org., Sett.) |  |
|  | One of the current trends in health-care design calls for cutting costs via substantially decreasing nonclinical spaces. There may be limitations for some clinics to provide a private quiet room allocated to this service. We also attempted to provide this service for another institution, but they were not able to allow access through a firewall from outside or provide other Internet options. | |  | calls for cutting costs (Sett.)  they were not able to allow access through a firewall from outside or provide other Internet options (Sett.) |  |
| Coyne^a^ experimental [39] | This time frame was chosen empirically, based on the amount of staff time available for follow-up calls. | |  | amount of staff time available (Org.) |  |
| Helpline Alz Ass East Massa [40] | The Alzheimer's Association through its network of helplines can provide valuable information to both informal and professional caregivers regarding the progression of the disease and its management. The Helpline should be recognized as a resource that may provide ongoing support through the duration of the disease. | | can provide valuable information (Org.) |  |  |
| Sabat^a^ [43] | Accomplishing all this through email correspondence is clearly possible. | | Accomplishing all this (…) is clearly possible (Prov.) |  |  |
| FITT-C [47–53] | We demonstrated the feasibility of recruiting and retaining distressed caregivers over the course of the 6-month intervention. Inclusion/exclusion criteria resulted in a group of caregivers reporting high levels of perceived burden and depressive symptoms. | | demonstrated the feasibility of recruiting and retaining distressed caregivers (Org.) |  |  |
| FITT-D [54] | A treatment manual and interventions guide were created, and a feasibility trial was then conducted in which 11 caregivers were enrolled in a three-month pre-test of the intervention. Following the three-month pre-test, further modifications to the intervention and outcome measures were made based on feedback from caregivers participating in the pre-test. However, these modifications were minimal (e.g., simplification of sample dialogue, addition of questions to assess key areas) because the intervention appeared to be feasible, was well received by the participants | | the intervention appeared to be feasible (Org.) |  |  |
|  | We found that caregivers who received the FITT were very satisfied with the treatment and the approach was feasible with this population. | | approach was feasible (Org.) |  |  |
|  | We trained two individuals who had no prior experience with dementia or caregiving in general. Therefore, we expect that the intervention could be easily learned and delivered by other healthcare professionals, such as nurses and social workers. | | the intervention could be easily learned and delivered (Org.) |  |  |
| NVAMP [55] | This study shows telephone calls that provide an opportunity for caregivers to talk may fulfill this confidant role for a number of caregivers. | | telephone calls that provide an opportunity for caregivers to talk (Org.) |  |  |
| ICSS [56–61] | Most participants had used email and had a regular email account for personal use. When using an email account in a Web-based portal, some were able to access the account and solve technical problems. For example, one caregiver said, “ I haven’t used it. I can’t find the address .” Others relied on their spouse or children to help solve related problems (e.g., going to the portal’s URL address, logging onto the e-mail account, and/or typing and sending e-mails). Because caregivers did not access the accounts frequently, they often forgot their user name and password or forgot that they had access to a support service. A caregiver said, “ I sometimes forgot to check the e-mails and the e-mails piled up .” | |  |  | some were able to access the account and solve technical problems (Sett.) |
|  | In this paper, the critical analysis of the exchanged e-mails demonstrated how clientcentred practice can be successfully applied in the new Internet-mediated intervention. | | can be successfully applied (Org.) |  |  |
|  | Delivering support services via ICT mediation seems to involve a complex interaction between the caregiver’s capacity to use the Internet and the caregiver’s needs for service. | | complex interaction between the caregiver’s capacity to use the Internet and the caregiver’s needs for service (Org., Con.) |  |  |
|  | The caregivers could not use the service even if they wanted to do so if access to the Internet was not available in the home or in the workplace or if there was no technical support. | |  | if access to the Internet was not available (Sett.)  no technical support (Sett.) |  |
|  | However, to compensate for a lack of nonverbal cues in the communication process, the person-centred communication needs adaptation in the asynchronous, text-based only context. | |  | lack of nonverbal cues (Prov.) |  |
|  | Despite the use of asynchronous, text-based communication strategies and the absence of visual and audio cues, the therapists and caregivers were able to develop meaningful working relationships that sustained the work of the therapy. | | able to develop meaningful working relationships (Prov.) | asynchronous, text-based communication strategies and the absence of visual and audio cues (Prov.) |  |
| InformCare [62–64] | The technical and logistical issues related to the development and functioning of the platform itself formed part of the anticipated challenges that our team had to address. The need to capture the diversity of care systems that exist in different countries, devise guidelines about how best to support family carers and offer an infrastructure that can be accessed and utilised by carers across Europe was not an easy task. | | technical and logistical issues related to the development and functioning of the platform itself formed part of the anticipated challenges (Org.) | was not an easy task (Org.) |  |
|  | Finally, the main barriers to use of the platform were time constraints, while technical problems and the complexity of the platform were reported as limiting factors by a minority of participants in Italy and Germany. | |  | technical problems and the complexity of the platform were reported as limiting factors by a minority of participants (Con.) |  |
| Laver^a^ [68,69] | It is feasible to offer dyadic interventions via telehealth and doing so reduces travel time and results in similar benefits for families. | | It is feasible to offer dyadic interventions via telehealth (Org.) |  |  |
|  | The results of this trial demonstrate that it was possible to adapt an evidence-based intervention which was designed to be offered in person and to offer the intervention using telehealth delivery. We found that telehealth delivery required less resource than home visits. | | it was possible to adapt (…) and to offer the intervention using telehealth delivery (Org.) |  | telehealth delivery required less resource (Org.) |
|  | Our results suggest that it is possible to assess, collaborate, problem solve, and personalize strategies with people with dementia and their care partners remotely using videoconferencing. | | Our results suggest that it is possible (…) remotely (Org.) |  |  |
|  | Therapists spent slightly less direct time with people receiving telehealth (although the difference was not statistically difference). Possible explanations include that there was less opportunity to model or role play via telehealth or there were fewer social conversations. | |  | less opportunity to model or role play via telehealth or there were fewer social conversations (Prov.) |  |
| RCTM [70–73] | Elements of the RCTM make this intervention amenable to dissemination and implementation. | | make this intervention amenable (Org.) |  |  |
| Dementelcoach [74–79] | The majority of the interviewed project leaders told us that the Dementelcoach concept was very clear to them and that they believed both interventions (Dementelcoach and STAR e-learning) are good additions to the MCSP. | | concept was very clear to them (Sett.) |  |  |
|  | Our study demonstrated the feasibility of this implementation process. Also, positive results were experienced by carers and other stakeholders in places where Dementelcoach and STAR e-learning was implemented successfully. | | demonstrated the feasibility (Org.) |  |  |
|  | Implementation of iMCSP proved to be feasible and to lead to a more varied group of participants (people with dementia, carers) utilizing the support. | | proved to be feasible (Org.) |  |  |

*Abbreviations*: FITT-C = Family Intervention: Telephone Tracking - Caregiver; FITT-D = Family Intervention: Telephone Tracking - Dementia; Helpline Alz Ass East Massa = Helpline of the Alzheimer's Association of Eastern Massachusetts.; ICSS = Internet-based Caregiver Support Service; NVAMP = Nurse Video With Assisted Modeling Program; Org. = Organization; Prov. = Provider; RCTM = Residential Care Transition Module; Sett. = Setting

*Note*: ^a^When no name is reported, the name of the first author was assigned to the intervention.

## Table S7: Analysis matrix for ‘fidelity’

| **Original definition of ‘fidelity’ according to Proctor et al. [17]:**  “*Fidelity* is defined as the degree to which an intervention was implemented as it was prescribed in the original protocol or as it was intended by the program developers (Dusenbury et al. 2003; Rabin et al. 2008).” | | **Adaptation of the definition:**  *Fidelity* is the degree to which an technology-based counselling intervention was implemented as it was prescribed in the original protocol or as it was intended by the program developers. | | |
| --- | --- | --- | --- | --- |
| **Intervention^a^** | **Quote** | | **Dimensions of ‘fidelity’ (level of analysis)** | |
|  |  |  | **Formalisation of intervention** | **Quality assurance in delivering the intervention** |
| Admiral Nurse Dementia Helpline [29–32] | During calls to the helpline, nurses follow the Admiral Nurse assessment framework (Harrison Dening 2010), a holistic, evidence-based tool that considers the biopsychosocial needs ot the person with dementia and their family or informal carers. | | nurses follow the Admiral Nurse assessment framework (Prov., Org.) |  |
| CANDID [37] | Senior medical supervision of such a service is vital, and information systems were developed to ensure adequate record-keeping and to assist medical review of advice given. | |  | Senior medical supervision (…) ensure adequate record-keeping (…) medical review of advice given (Org.) |
| Care Consultation / Care Consultation Plus^b^ [38] | In addition, while fidelity checks were conducted with 20 % of all calls, variability in the delivery style of care consultations was a common observation. | |  | fidelity checks were conducted (…) variability in the delivery style (…) was a common observation (Org.) |
| Helpline Alz Ass East Massa [40] | The National Alzheimer's Association should update their resource and policy manual for use by all chapters. | | resource and policy manual (Org.) |  |
| FITT-C [47–53] | All follow-up contacts will follow a similar protocol: 1) introduction — identify purpose of call; 2) assessment of current status — identify positive and negative changes since last contact; 3) assessment of key areas — note any changes in each key area of functioning (i.e., health, functioning, mood, social support, and family life), repeated review of each of these key areas during every call, implicitly reinforces the need to appraise and reappraise these issues; 4) review of other issues — identify other issues that could be problematic; 5) intervention — provide support and assistance to help caregivers solve problems and utilize family resources; and 6) continuing education — provide an opportunity for caregivers to ask questions about dementia or the care recipient. | | contacts will follow a similar protocol (Prov., Org.) |  |
|  | Throughout the study, therapists in each condition received weekly 1-hour group supervision with neuropsychologists with expertise in dementia and caregiving (GT & JD). The goal of these sessions was to discuss specific cases, to ensure competent implementation of the treatment, and to reinforce adherence to the intervention. Periodically, consultant experts attended the group supervision to offer continuing education about dementia and to address any issues that arose with specific participants. All telephone contacts were audiotaped, and a subset were reviewed during supervision sessions to ensure adherence and to better guide therapists' intervention strategies. Any deviations from the treatment protocol were brought to the therapist's attention for remediation. In addition, a randomly selected subgroup of tapes were reviewed by a trained research assistant, who rated adherence to the intervention and competence using standardized rating scales. | |  | weekly 1-hour group supervision (…) to ensure competent implementation of the treatment, and to reinforce adherence (Prov., Org.)  reviewed during supervision sessions to ensure adherence (Org.)  deviations from the treatment protocol were brought to the therapist's attention for remediation (Prov., Org.)  rated adherence to the intervention and competence using standardized rating scales (Org.) |
|  | Quality control was implemented by weekly supervision of both the FITT-C and TS therapists. | |  | Quality control was implemented by weekly supervision (Org.) |
| FITT-D [54] | Doctoral-level staff supervised therapists weekly to ensure adherence to the protocol and minimize drift. Sessions were audiotaped, and two raters reviewed 30 randomly selected telephone contacts and independently completed adherence and competence scales. Both therapists demonstrated competency and adhered to the treatment. | |  | supervised therapists weekly to ensure adherence to the protocol and minimize drift (…) Sessions were audiotaped, and two raters (…) independently completed adherence and competence scales (Org.) |
|  | Each contact followed a standardized treatment manual, involving assessment and individualized application of interventions to address mood, family functioning, social support and health. | | standardized treatment manual, involving assessment and individualized application of interventions (Prov., Org.) |  |
| InformCare [62–64] | Guidelines for moderators were developed before the program began, based on a review of key recommendations in the field of online counseling. | | Guidelines for moderators were developed (Org) |  |
| Laver^a^ [68,69] | Regular (fortnightly) meetings were held with interventionists to discuss cases and treatment plans and monitor fidelity to the intervention. Therapists also kept treatment notes, time logs, and recorded the dates in which key components of the program were offered. | |  | meetings were held with interventionists to (…) monitor fidelity to the intervention (Prov., Org.) |

*Abbreviations*: CANDID = Counselling and Diagnosis in Dementia; FITT-C = Family Intervention: Telephone Tracking – Caregiver; FITT-D = Family Intervention: Telephone Tracking – Dementia; Helpline Alz Ass East Massa = Helpline of the Alzheimer's Association of Eastern Massachusetts; Org. = Organization; Prov. = Provider

*Notes*: ^a^When no name is reported, the name of the first author was assigned to the intervention.

^b^Assignment of the quote(s) to experimental and comparator intervention not possible, we assume, the information is applicable for both interventions.

## Table S8: Analysis matrix for ‘implementation cost’

| **Original definition of ‘implementation cost’ according to Proctor et al. [17]:**  “Cost *(incremental or implementation cost)* is defined as the cost impact of an implementation effort.” | | **Adaptation of the definition:**  *Implementation cost* is the cost impact of an implementation effort. | | | |
| --- | --- | --- | --- | --- | --- |
| **Intervention^a^** | **Quote** | | **Dimensions of ‘implementation cost’ (level of analysis)** | | |
|  |  |  | **Cost impact of delivery because of complexity of intervention** | **Cost impact of implementation because of complexity of implementation strategy** | **Cost impact because of varying complexity of settings** |
| ALZ i-Connect [36] | The only costs incurred by the chapter have been the purchase of several flat screen TVs and cameras which the Association was able to fund in lieu of mileage costs previously spent. | | only costs incurred … was able to fund in lieu of mileage costs previously spent (Org.) |  |  |
| InformCare [62–64] | Resources did not allow us to expand the scope of the platform to those countries not included in the original. | |  |  | Resources did not allow us to expand (Org.) |
| Dementelcoach [74–79] | The cost of the Dementelcoach intervention connected to a Meeting Centre is 575 euro per individual. | | 575 euro per individual (Org.) |  | cost of the Dementel-coach intervention connected to a Meeting Centre (Org.) |
|  | With regard to human and financial resources, appointing a motivated and creative project leader, who has enough time to invest in the project, was important. Funds available for the project facilitated creating this time or hiring personnel to help with the implementation. Project leaders who did not have these funds emphasized this was an impeding factor. Another problem was that the Dementelcoach cooperation lacked the financial resources to provide the amount of coaching to the Meeting Centres they would have liked to guide and stimulate the implementation. | |  | Funds available for the project facilitated creating this time or hiring personnel to help with the implementation. Project leaders who did not have these funds emphasized this was an impeding factor. (Org.)  Dementelcoach cooperation lacked the financial resources to provide the amount of coaching … to guide and stimulate the implementation (Org.) |  |
|  | Two Meeting Centres did not succeed in raising funds for Dementelcoach during the time of the project. | |  |  | Two Meeting Centres did not succeed in raising funds for Dementelcoach (Org.) |

*Abbreviation*: Org. = Organization

## Table S9: Analysis matrix for ‘penetration’

| **Original definition of ‘penetration’ according to Proctor et al. [17]:**  “*Penetration* is defined as the integration of a practice within a service setting and its subsystems.” | | **Adaptation of the definition:**  *Penetration* is defined as the integration of a technology-based counselling intervention within a service setting. | | | |
| --- | --- | --- | --- | --- | --- |
| **Intervention^a^** | **Quote** | | **Dimensions of penetration (level of analysis)** | | |
|  |  |  | **Collaboration with stakeholders** | **Access to the service** | **Spread** |
| Admiral Nurse Dementia Helpline [29–32] | Admiral Nurses provide advice on referrals to other appropriate services and liaise with other health professionals on behalf of the family. | | Provide advice on referrals to other appropriate services (Org.) |  |  |
| ADS helpline [33] | Most people had rung the ADS following a suggestion from their general practitioner (GP) (…) | |  | Had rung the ADS following a suggestion from their general practitioner (Sett.) |  |
| Alzheimer helpline [34,35] | Nur 1% der Anrufer bekamen die Nummer des Alzheimer-Telefons von ihrem Arzt. | |  | Nur 1% (…) bekamen die Nummer (…) von ihrem Arzt (Org., Sett.) |  |
|  | *Only 1% of the callers got the number of the Alzheimer helpline from their physician.*  *(translated by authors)* | |  | *Only 1% (…) got the number (…) from their physician (Org., Sett.)* |  |
|  | Die Vermittlung von Anlaufstellen vor Ort ist wichtiger Bestandteil der Gespräche am Alzheimer-Telefon. | | Vermittlung von Anlaufstellen ist ein wichtiger Bestandteil (Org.) |  |  |
|  | *The referral to local services is an important part of the conversations on the Alzheimer helpline.*  *(translated by authors)* | | *referral to local services is an important part (Org.)* |  |  |
|  | (…) dem einzigen bundesweit agierenden, vom Bundesministerium für Familie, Senioren, Frauen und Jugend finanziell unterstützten, demenzspezifischen, telefonischen Beratungsangebot Deutschlands. | |  |  | Dem einzigen bundesweit agierenden (…) Beratungsangebot Deutschlands (Org., Sett.) |
|  | *(...) the only nationwide operating dementia-specific telephone counselling service in Germany, financially supported by the Federal Ministry for Family Affairs, Senior Citizens, Women and Youth.*  *(translated by authors)* | |  |  | *the only nationwide operating (…) counselling service in Germany (Org., Sett.)* |
| ALZ i-Connect [36] | The Alzheimer’s Association Cleveland Area Chapter has maintained ongoing efforts to integrate innovative support services alongside traditional primary care for people with dementia. For several years, the chapter collaborated with area assessment center programs in northeast Ohio by placing an Association staff person at the center to be called upon by the medical team as a resource to patients and their families who were interested in learning more about the disease and available services. | | Collaborated with area assessment center programs (Org., Sett.) |  |  |
|  | It is imperative that organizations like the Alzheimer’s Association seek to collaborate and partner with the healthcare community to ensure that every individual who receives a diagnosis of Alzheimer’s disease or another form of dementia receives the information and referral services they need. The ALZ i-Connect program has proven to be just such an innovative collaborative model. The program has allowed the Cleveland Area Chapter to interface with 2 key assessment center programs by providing the complementary support that is needed to educate their patients about Alzheimer’s disease and the supportive services available from the Association in an efficient and effective way. | | Seek to collaborate and partner with healthcare community (Org., Sett.) |  |  |
|  | The solution was the creation of ALZ i-Connect, a unique approach to connect individuals who have just received a diagnosis to important information about memory loss and the Association’s free services before they even leave their physician’s office. | |  | To connect individuals (…) to the (…) services before they even leave their physician’s office (Org., Sett.) |  |
| CANDID [37] | The clinic is a national referral service and attracts patients from throughout the UK, and by contrast to the small numbers of cases seen in local services, more than 150 new referrals are seen each year, with over 500 patients in ongoing follow-up. | |  | more than 150 new referrals are seen each year (Org., Sett.) | patients from throughout the UK (Org., Sett.) |
|  | To develop a communication network to coordinate the activity of other services providing care for this group of patients. | | To develop a communication network to coordinate the activity of other services (Org.) |  |  |
|  | A broad range of carers and professionals have used the service. It was an intention of the service to provide a source of expert advice to professionals; however, that only 5% of calls were from doctors and only 13% from nurses and social workers suggests that this aim has not been achieved. | |  | broad range of carers and professionals have used the service (Org., Sett.)  only 5% of calls were from doctors and only 13% from nurses and social workers (Org., Sett.) |  |
|  | Over the 2-year period CANDID organized and ran four teaching courses, two professional fora and a conference on Pick's disease attended by a total of 287 people. Regular teaching commitments were established with three university nursing courses and CANDID was coopted to four national committees. Additionally, presentations on CANDID were given at 17 national meetings and conferences and at one international meeting. There was also publicity from appearances on national and local radio programmes and regional television. | | organized and ran four teaching courses, two professional for a and a conference (Org.)  Regular teaching commitments (Org.)  was coopted to four national committees (Org.) |  |  |
|  | CANDID represents a new concept in providing care for a group of patients with rare diseases. Since its inception it has had a rapid uptake with carers, although doctors and other professionals have made relatively less use of the service than was originally anticipated. | |  | rapid uptake with carers, although doctors and other professionals have made relatively less use of the service (Org., Sett.) |  |
|  | The service has become rapidly accepted and used by families of patients and members of the public. Healthcare professionals have made less use of the service than anticipated, but it is hoped that this will increase as information about the service becomes disseminated. | |  | used by families of patients and members of the public (Org., Sett.)  Healthcare professionals have made less use of the service (Org., Sett.) |  |
|  | CANDID is such a service, which serves a small population of patients and their carers who are distributed across the UK; its telemedicine model also divers from others, enclosing the caregiver/ patient in a loop with the service (Fig. 2). | |  |  | Who are distributed across the UK (Org., Sett.) |
| Helpline Alz Ass East Massa [40] | The local chapter of the Alzheimer's Disease and Related Disorders Association of Eastern Massachusetts is one of more than 200 chapters nationally. The Boston-based chapter is amongst the top 10 chapters in the nation based on annual budget, which is in the area of $600,000. | |  |  | Is one of more than 200 chapters nationally (Org., Sett.) |
|  | About 12 percent (12) of the callers found out about the helpline through the phone book, 13 percent (13) from a social worker, 11 percent (11) through a newspaper and 9 percent (9) by a physician. About 6 percent (6) of the callers found out about the helpline through other sources such as through work or by making a donation to the National Association. | |  | Found out (…) through the phone book, (….) from a social worker), (…) through a newspaper (…) by a physician (Org., Sett.) |  |
| InformCare [62–64] | This social innovation was constituted by the new InformCare Web platform, which was intended to act as a first point of access to a variety of information, education, and social support opportunities at the country level for family caregivers, as well as an opportunity for formal services and nonprofit organizations in the field. | | an opportunity for formal services and nonprofit organizations (Org., Sett.) | to act as a first point access (…) for family caregivers (Org., Sett.) |  |
|  | since the platform has been accessible since mid-2015 in 27 EU countries via the Eurocarers website [60]. It includes 32 national versions, with some countries having more than one official language, and more than 2500 Web pages in the information resources area, which are publicly available and tailored to country characteristics. According to the availability and resources of national nonprofit organizations appointed in each country, a selection of interactive services may have been activated for national caregivers as well. | | availability and resources of national nonprofit organizations appointed in each country (Org., Sett.) |  | and more than 2500 web pages in the information resources area (…) and tailored to country characteristics (Org., Sett.) |
|  | In this respect, the InformCare (Eurocarers, Brussels, Belgium) Web platform23 was created to address this gap, and constituted the first social innovation in Europe to be widely and freely available since mid-2015 in 32 versions, covering 27 countries with more than 2500 Web pages in 23 official languages of the EU. | |  |  | Covering 27 countries with more than 2500 web pages in 23 official languages of the EU (Org., Sett.) |
| Link2Care [65] | Through the combined efforts of three innovative programs— FamilyCaregiverAlliance (fca), which provided its successful service model, information clearing house and website; the University of Wisconsin’s Comprehensive Health Enhancement Support System (chess); and the California Caregiver Resource Centers’ (crcs) statewide service delivery system—Link2Care offered a response to the growing information and support needs of caregivers of adults with cognitive impairment. | | Through the combined efforts of three innovative programs (Sett.) |  |  |
|  | The program complements services delivered by the crcs and provides an enriched information and support environment. Once a family caregiver (or friend or partner) completes the initial crc intake screening, the person is invited to register for Link2Care online. | | The program complements services (Org., Sett.) |  |  |
|  | Initially offered as a demonstration program in five Caregiver Resource Centers covering 40 counties of the state, Link2Care is now offered throughout California. | |  |  | is now offerend throughouth California (Org., Sett.) |
| De Cola^a^ [67] | included a telehealth system within a family-centred care programme | | Within a family-centred care programme (Org., Sett.) |  |  |
|  | The telehealth system used in our program included a box installed at the nursing home ‘Casa Pia’ of Messina, and connected via internet to the Laboratory of Telemedicine of the IRCCS Centro Neurolesi ‘Bonino-Pulejo’ of Messina. | | Installed at the nursing home (…) in Messina (Org., Sett.) |  |  |
|  | Differently from previous work, our subjects were not tele-monitored within their own homes or hospitals, but within an integrated family-centred programme. | | within an integrated family-centred programme (Org., Sett.) |  |  |
| Dementelcoach [74–79] | Another important organizational condition proved to be being located in an active region, i.e a region where innovative care initiatives are happening, and opportunities for starting new ones are available, as well as collaboration exists between care and welfare organisations. Being part of a care and welfare network proved to be a facilitating factor as it helped to reach the target group. | | Opportunities for starting new ones (…), as well as collaboration exists (Org., Sett.)  Being part of a care and welfare network (Org., Sett.) |  |  |
|  | Implementation of dementelcoach and STAR e-Learning: The implementation of both new interventions took place at 8 Meeting Centres according to a stepwise plan. | | Took place at 8 meeting centres (Org., Sett.) |  |  |
|  | Competition: In some regions the new interventions, but especially Dementelcoach, were seen as competition by case-managers, and therefore met resistance. This was an impeding factor in both the preparation and starting phases of the implementation. | | Were seen as competition (Sett.) |  |  |
| Nomura^a^ [80] | The monthly activity-based programme commenced in July 2000 at a centre for the elderly located in the centre of the town. | | At a centre for elderly (Org., Sett.) |  |  |

*Abbreviations*: ADS = Alzheimer's Disease Society; CANDID = Counselling and Diagnosis in Dementia; Helpline Alz Ass East Massa = Helpline of the Alzheimer's Association of Eastern Massachusetts; Org. = Organization; Sett. = Setting

*Note*: ^a^When no name is reported, the name of the first author was assigned to the intervention.

## Table S10: Analysis matrix for ‘sustainability’

| **Original definition of ‘sustainability’ according to Proctor et al. [17]:**  “*Sustainability* is defined as the extent to which a newly implemented treatment is maintained or institutionalized within a service setting’s ongoing, stable operations.” | | **Adaptation of the definition:**  *Sustainability* is defined as the extent to which an implemented technology-based counselling intervention is maintained or institutionalized within a organization’s ongoing, stable operations. | | | | |
| --- | --- | --- | --- | --- | --- | --- |
| **Intervention^a^** | **Quote** | | **Dimensions of ‘sustainability’ (level of analysis)** | | | |
|  |  |  | **Routinisation** | **Passage** | **Incorporation** | |
| Admiral Nurse Dementia Helpline [29–32] | While telehealth may be a new concept for some healthcare settings, it has been available for over 15 years for families living with dementia via the Dementia UK Admiral Nurse Dementia Helpline, which handles an average of 20,000 contacts each year. Dementia UK has established systems and processes to mitigate some of the previously mentioned challenges to delivering telehealth. | | for over 15 years (Org.); handles an average of 20,000 contacts each year (Org.) | To mitigate some of the previously mentioned challenges (Org.) | has been available … via the Dementia UK Admiral Nurse Dementia Helpline (Org.) | |
|  | Dementia UK is a charity committed to helping families face dementia. (…) The charity’s Admiral Nurse dementia telephone helpline is a key part of its work. | |  |  | key part (Org.) | |
| ADS helpline [33] | The Alzheimer's Disease Society (ADS) established telephone helplines some time ago to provide support, advice and information to carers of people with dementia. Times change, and over the last few years the ADS has recorded a small but increasing number of calls from people with dementia themselves, asking for help. | | some time ago (Org.);  over the last few years (Org.) | a small but increasing number of calls from people with dementia themselves (Org.) |  | |
| Alzheimer helpline [34,35] | Seitdem haben fast 30000 Anrufer dieses Angebot wahrgenommen (…). | | fast 30000 Anrufer (Org.) |  |  | |
|  | *Since then, almost 30,000 callers have taken up this offer (...).*  *(translated by authors)* | | *almost 30,000 callers (Org.)* |  |  | |
|  | Im Jahr 2006 haben sich 5449 Anrufer an das Alzheimer-Telefon gewandt. | | 5449 Anrufer an das Alzheimer-Telefon gewandt (Org.) |  |  | |
|  | *In 2006, 5,449 callers contacted the Alzheimer helpline.*  *(translated by authors)* | | *5,449 callers contacted the Alzheimer helpline (Org.)* |  |  | |
|  | Im Jahr 2006 gaben 20% an, dass ihnen die Nummer bekannt war und sie nicht mehr sagen konnten, woher. Das ist nach fünf Jahren ein guter Erfolg für die Bekanntmachung des Angebots. | | nach fünf Jahren ein guter Erfolg für die Bekanntmachung (Org.) |  |  | |
|  | *In 2006, 20% said they knew the number and could no longer say where from. After five years, this is a good success for publicising the offer.*  *(translated by authors)* | | *After five years, this is a good success for publicising (Org.)* |  |  | |
|  | Auch der Bedarf an Beratung und Information ist nach wie vor vorhanden, was die stetigen Anruferzahlen zeigen, sodass das Alzheimer-Telefon auch nach fünf Jahren ein wichtiges Angebot für die Menschen mit Demenz und ihre Angehörigen bleibt. | | nach fünf Jahren (Org.) stetige Anruferzahlen (Org.) |  |  | |
|  | *The need for counselling and information is also still there, which is shown by the steady number of callers, so that the Alzheimer helpline remains an important service for people with dementia and their relatives even after five years.*  *(translated by authors)* | | *after five years (Org.)*  *steady number of callers (Org.)* |  |  | |
|  | Das Alzheimer-Telefon der Deutschen Alzheimer Gesellschaft wurde im Januar 2002 (...) eröffnet. | | Eröffnung im Januar 2002 (Org.) |  |  | |
|  | *The Alzheimer helpline of the German Alzheimer's Association was opened (...) in January 2002.*  *(translated by authors)* | | *was opened (...) in January 2002 (Org.)* |  |  | |
|  | Seit 15 Jahren existiert das Alzheimer - Telefon der Deutschen Alzheimer Gesellschaft e.V. (DAlzG) als bundesweites psychosoziales Beratungsangebot und hat sich als wichtige Säule bei der Unterstützung von Menschen mit Demenz und ihren Familien etabliert. | | Existiert seit 15 Jahren (Org.)  als wichtige Säule  etabliert (Org.) |  | Alzheimer-Telefon der Deutschen Alzheimer Gesellschaft e.V. (Org.) | |
|  | *The Alzheimer helpline of the German Alzheimer's Association (DAlzG) has existed for 15 years as a nationwide psychosocial counselling service and has established itself as an important pillar in the support of people with dementia and their families.*  *(translated by authors)* | | *has existed for 15 years (Org.)*  *has established itself as an important pillar (Org.)* |  | *Alzheimer helpline of the German Alzheimer's Association (DAlzG) (Org.)* | |
|  | Eine Stärke der Analyse ist, dass Daten verarbeitet wurden, die auf dem einzigen mit institutioneller Förderung bundesweit agierenden, telefonischen Beratungsangebot für Angehörige eines Demenzbetroffenen beruhen. Es wird vermutet, dass es dadurch auch das in Deutschland am meisten genutzte Beratungstelefon für Fragen rund um das Thema Demenz ist. | |  |  | bundesweit agierenden (Org.) | |
|  | *A strength of the analysis is that data was processed that is based on the only telephone counselling service for relatives of someone affected by dementia that operates nationwide with institutional funding. It is assumed that this also makes it the most used counselling telephone in Germany for questions about dementia.*  *(translated by authors)* | |  |  | *operates nationwide (Org.)* | |
| ALZ i-Connect [36] | This model can be a complementary approach to the currently available phone-based helpline and provides an easy way for individuals at remote centers to connect with the association. | |  |  | Provides an easy (…) to connect with the association (Org.) | |
| CANDID [37] | This article presents an evaluation of the first 2 years of the service. | | first 2 years (Org.) |  |  |  |
|  | We are currently developing proposals to expand CANDID on both a national and European level. Local CANDID offices would be able to take calls from carers in several districts, with information technology used to route complex queries back to the central CANDID hub. | |  | Proposal to expand the intervention on a national and European level (Org.) |  |  |
|  | The CANDID (Counselling and Diagnosis in Dementia) service was launched in February 1995 | | Launched in February 1995 (Org.) |  |  |  |
| Care Consultation [38] | already-established telephone support Helpline | | already-established (Org.) |  |  |  |
|  | Yearly, the Helpline receives between 290,000 and 300,000 calls, which averages to about 800 calls per day. | | Yearly (…) receives between 290,000 and 300,000 calls (Org.) |  |  |  |
| Helpline Alz Ass East Massa [40] | The helpline represents 20 percent of the organization's activities | |  |  | 20 percent of the organization's activities (Org.) |  |
|  | The helpline receives approximately 4,000 calls per year with a peak week averaging 86 calls. | | receives approximately 4,000 calls per year (Org.) |  |  |  |
| ODCC [42] | The association for people with dementia and their families in Okayama, Japan, is a local public interest incorporated association, which established a telephone support system for dementia, named ODCC, entrusted by both of Okayama city and Okayama prefecture in June 2011. | | established … in June 2011 (Org.) |  |  |  |
| FITT-C [47–53] | The next step in moving the FITT-C into the community is to determine its cost-effectiveness, and to examine whether the FITT-C can perform under “real-world” conditions in a community implementation trial. | |  | The next step … to examine whether the FITT-C can perform under “real-world” conditions (Org.) |  |  |
| InformCare [62–64] | This constitutes a remarkable, concrete added value of this research, since the platform has been accessible since mid-2015 in 27 EU countries via the Eurocarers website [60]. | | accessible since mid-2015 (Org.);  in 27 EU countries (Org.) |  | via the Eurocarers website (Org.) |  |
|  | The platform has been ‘open for business’ since mid-2015 via the Eurocarers website. | | since mid-2015 (Org.) |  | via the Eurocarers website (Org.) |  |
|  | A key question relates to the long-term maintenance and sustainability of the InformCare platform. The platform demands a significant amount of ongoing support and investment to keep it up to date and populated with relevant information, accessible 24/7 by carers and other relevant stakeholders in 32 languages. | | A key question … long-term maintenance  significant amount of ongoing … investment (Org.) |  |  |  |
|  | In this respect, the InformCare (Eurocarers, Brussels, Belgium) Web platform23 was created to address this gap, and constituted the first social innovation in Europe to be widely and freely available since mid-2015 in 32 versions, covering 27 countries with more than 2500 Web pages in 23 official languages of the EU. | | since mid-2015 (Org.);  32 versions, covering 27 countries (Org.) |  |  |  |
| Link2Care [65] | Initially offered as a demonstration program in five Caregiver Resource Centers covering 40 counties of the state, Link2Care is now offered throughout California. | | covering 40 countries (Org.) |  | Initially offered as a demonstration program, now offered throughout California (Org.) |  |
|  | To date, more than 700 caregivers have been enrolled | | more than 700 caregivers have been enrolled (Org.) |  |  |  |
| Online Coaching Program [66] | The Online Coaching Program was implemented as a pilot project in January 2006 by the Greater Cincinnati Chapter of the Alzheimer's Association. | | a pilot project in January 2006 (Org.) |  |  |  |
|  | Since alzcoaching.org began in January 2006, 121 caregivers have enrolled in the program. | | 121 caregivers have enrolled (Org.) |  |  |  |
| De Cola^a^ [67] | Unfortunately, this care programme was provided by our local Government for only two years, and we were not able to recruit more than 18 elderly people meeting our inclusion criteria. | | for only two years (Org.) | was provided by our local government for only two years (Admin.) |  |  |

*Abbreviations*: Admin. = Administration; ADS = Alzheimer's Disease Society; CANDID = Counselling and Diagnosis in Dementia; FITT-C = Family Intervention: Telephone Tracking – Caregiver; Helpline Alz Ass East Massa = Helpline of the Alzheimer's Association of Eastern Massachusetts; ODCC = Okayama Dementia Call Center; Org. = Organization

*Note*: ^a^When no name is reported, the name of the first author was assigned to the intervention.
